# Catalytic mechanism of *trans*-2-enoyl-CoA reductases in the fatty acid elongation cycle and its cooperative action with fatty acid elongases

**DOI:** 10.1016/j.jbc.2024.105656

**Published:** 2024-01-13

**Authors:** Ryoya Kato, Yuka Takenaka, Yusuke Ohno, Akio Kihara

**Affiliations:** Faculty of Pharmaceutical Sciences, Hokkaido University, Sapporo, Japan

**Keywords:** ceramide, enzyme catalysis, fatty acid, fatty acid elongation cycle, fatty acid metabolism, lipid, sphingolipid, *trans*-2-enoyl-CoA reductase, very-long-chain fatty acid, yeast

## Abstract

The fatty acid (FA) elongation cycle produces very-long-chain FAs with ≥C21, which have unique physiological functions. *Trans*-2-enoyl-CoA reductases (yeast, Tsc13; mammals, TECR) catalyze the reduction reactions in the fourth step of the FA elongation cycle and in the sphingosine degradation pathway. However, their catalytic residues and coordinated action in the FA elongation cycle complex are unknown. To reveal these, we generated and analyzed Ala-substituted mutants of 15 residues of Tsc13. An *in vitro* FA elongation assay showed that nine of these mutants were less active than WT protein, with E91A and Y256A being the least active. Growth complementation analysis, measurement of ceramide levels, and deuterium-sphingosine labeling revealed that the function of the E91A mutant was substantially impaired *in vivo*. In addition, we found that the activity of FA elongases, which catalyze the first step of the FA elongation cycle, were reduced in the absence of Tsc13. Similar results were observed in Tsc13 E91A-expressing cells, which is attributable to reduced interaction between the Tsc13 E91A mutant and the FA elongases Elo2/Elo3. Finally, we found that E94A and Y248A mutants of human TECR, which correspond to E91A and Y256A mutants of Tsc13, showed reduced and almost no activity, respectively. Based on these results and the predicted three-dimensional structure of Tsc13, we speculate that Tyr256/Tyr248 of Tsc13/TECR is the catalytic residue that supplies a proton to *trans*-2-enoyl-CoAs. Our findings provide a clue concerning the catalytic mechanism of Tsc13/TECR and the coordinated action in the FA elongation cycle complex.

Fatty acids (FAs) are major components of lipids. The group includes diverse molecular species differing in carbon-chain length and the number and position of double bonds. They are classified based on chain length as short-chain (C2–C4), medium-chain (C5–C10), long-chain (C11–C20), or very-long-chain (VLC; ≥C21) FAs, and C16–C20 long-chain FAs are the most abundant in mammalian lipids (1, 2, 3, 4). Although the quantities of VLCFAs in mammalian lipids are much lower than those of long-chain FAs, they play unique physiological roles in functions such as myelin formation and maintenance, skin barrier formation, retinal function, spermatogenesis, and tear film stabilization (3, 4, 5, 6, 7, 8, 9).

Type I FA synthases (FASs) produce predominantly C16:0 FA in mammals and C16:0-CoA and C18:0-CoA in yeast (10, 11, 12). Some of the long-chain FAs produced by the FASs or absorbed from the diet are elongated to VLCFAs *via* the FA elongation cycle in the endoplasmic reticulum (ER) after conversion to acyl-CoAs (3, 4). The cycle consists of four reactions (condensation, reduction, dehydration, and reduction) and produces acyl-CoAs that are elongated by two carbons per cycle (Fig. 1*A*). The first reaction is the condensation of acyl-CoA (carbon-chain length: n) with a malonyl-CoA to form 3-ketoacyl-CoA (n + 2). This is then reduced to (*R*) 3-hydroxy (3-OH) acyl-CoA (n + 2), followed by dehydration to *trans*-2-enoyl-CoA (n + 2). This then undergoes a second reduction to produce acyl-CoA (n + 2). The enzymes catalyzing each reaction are conserved across a wide range of eukaryotes, including yeast, plants, and mammals (3, 4, 13), and those in yeast and mammals are as follows: FA elongases ELOVL1–7 (mammals) and Elo1–3 (yeast) (3, 4, 14, 15); 3-ketoacyl-CoA reductases KAR (also called HSD17B12; mammals) and Ifa38 (yeast) (16, 17); 3-OH acyl-CoA dehydratases HACD1 and HACD2 (mammals) and Phs1 (yeast) (18, 19, 20); and *trans*-2-enoyl-CoA reductases TECR (also called GPSN2 and TER; mammals) and Tsc13 (yeast) (17, 21).

**Figure 1:**
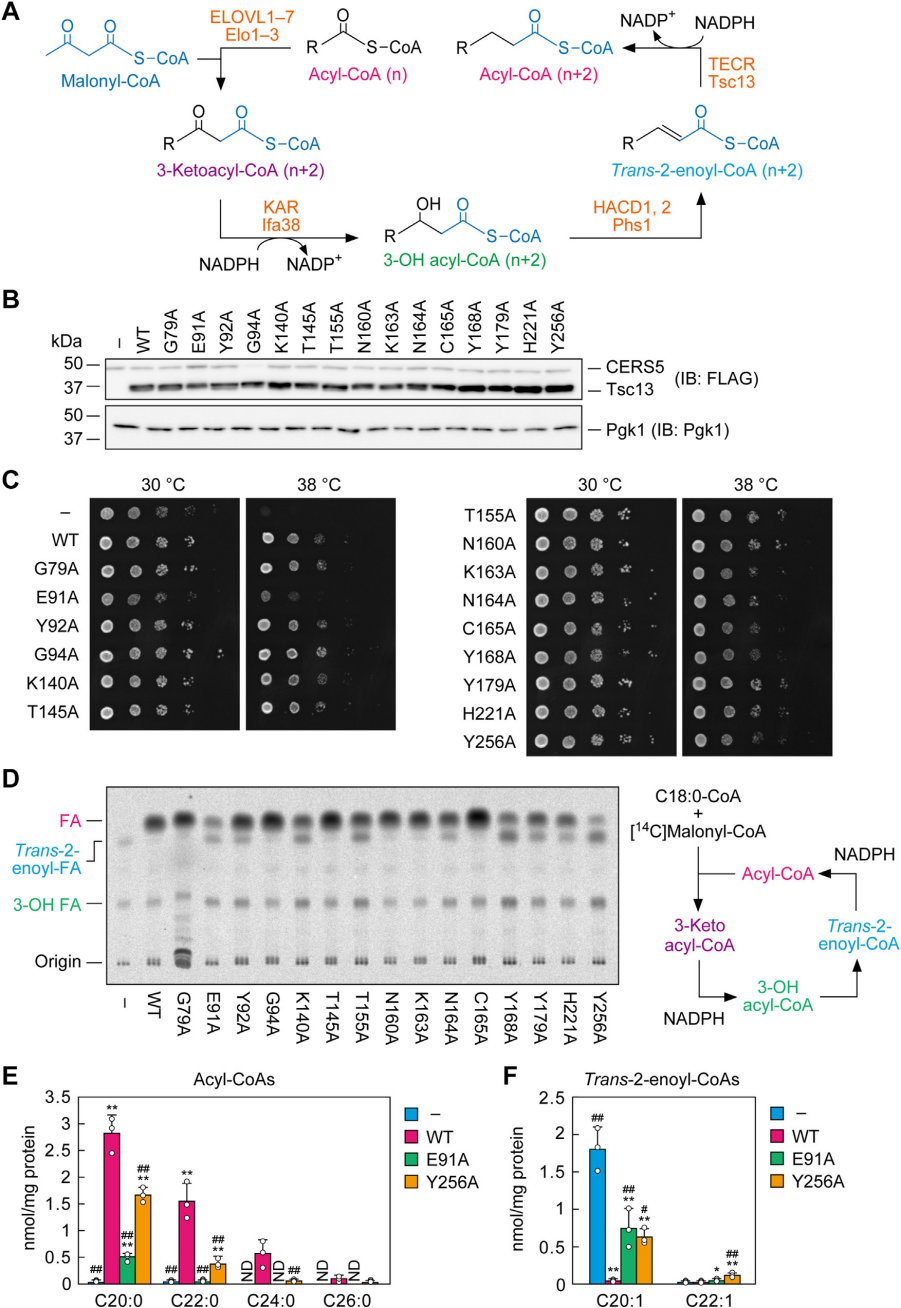
**Identification of amino acid residues important for Tsc13 activity.***A*, schematic representation of the FA elongation cycle. The structures of the substrates/products for the four reactions in the FA elongation cycle (condensation, reduction, dehydration, and reduction) and the names of the mammalian and yeast proteins involved in each reaction are shown. *B*–*F*, ABY80 (*tsc13*Δ/*CERS5*) cells harboring the vector or the plasmid encoding *3×FLAG-TSC13* (WT or mutants) were grown to early-log phase at 30 °C. *B*, total cell lysates were prepared from the cells and separated *via* SDS-PAGE, followed by immunoblotting with anti-FLAG and anti-Pgk1 (loading control) antibodies. *C*, cells were diluted to *A*_600_ = 1.5 and further serially diluted 10-fold. Cells were spotted onto plates of SC –His –Ura and incubated at 30 °C or 38 °C. *D*–*F*, membrane fractions were prepared and subjected to an *in vitro* FA elongation assay. *D*, membrane fractions (10 μg) were incubated with 20 μM C18:0-CoA, 27.3 μM [^14^C]malonylCoA, and 1 mM NADPH at 37 °C for 30 min. Acyl-CoAs were alkaline-hydrolyzed to FAs, neutralized, extracted, separated *via* TLC, and detected using a bioimaging analyzer BAS-2500. The schematic diagram of the assay system is shown on the *right*. *E* and *F*, the total membrane fraction (10 μg) was incubated with 10 μM C18:0-CoA, 100 μM [^13^C]malonylCoA, and 1 mM NADPH at 37 °C for 30 min. Acyl-CoAs were alkaline-hydrolyzed to FAs, neutralized, and extracted. The resulting FAs were derivatized with AMP amide and quantified *via* LC-MS/MS. Values are the quantities of acyl-CoAs (*E*) and *trans*-2-enoyl-CoAs (*F*) with the respective chain length/degree of unsaturation and are the means + SD from three independent experiments. Statistically significant differences are indicated (Dunnett’s test; ∗*p* < 0.05, ∗∗*p* < 0.01 [*versus* vector control]; ^#^*p* < 0.05; ^##^*p* < 0.01 [*versus* WT]). IB, immunoblotting.

Saturated and monounsaturated VLCFAs produced *via* the FA elongation cycle are mainly used for the synthesis of sphingolipids. The hydrophobic backbone of sphingolipids is ceramide, composed of a long-chain base (LCB) and an FA (22). The most abundant LCB in mammals is sphingosine, which has a *trans* double bond at C4. In most mammalian tissues, sphingolipids are composed of C16–C24 saturated FAs (C16:0–C24:0) or monounsaturated FAs (mainly C24:1). The percentages of sphingolipids containing VLCFAs vary among tissues (20–80%) (4, 23). Yeast, however, does not have sphingosine but instead phytosphingosine with a hydroxyl group at C4 as the major LCB (24). The FA moiety of yeast sphingolipids is mostly 2-hydroxy (2-OH) C26:0 FA (25). Since the substrates of yeast ceramide synthase are VLC acyl-CoAs but not long-chain acyl-CoAs (26), aberration of the FA elongation cycle, such as deletion of the *PHS1* (*phs1*Δ) or *TSC13* (*tsc13*Δ), causes defects in ceramide/sphingolipid synthesis, which are lethal (18, 21, 27). Of the FA elongases Elo1–3, Elo2 and Elo3 function redundantly in the production of VLCFAs (15). Although deletion mutants of one of these only (*elo2*Δ and *elo3*Δ) grow normally, simultaneous disruption of *ELO2* and *ELO3* (*elo2*Δ *elo3*Δ) is lethal (28, 29). In mammals, aberration of the FA elongation cycle also causes lethality. For example, KO mice of *Kar* or *Tecr* are embryonic lethal due to impaired organogenesis (30, 31). While a single KO of *ELOVLs* and *HACDs* does not cause embryonic lethality due to the existence of multiple isozymes (7, 32, 33, 34, 35, 36), some of these genes are known to be responsible for hereditary diseases in humans. For example, *ELOVL1* or *ELOVL4* mutations cause neurocutaneous disorders or macular degenerative diseases (37, 38, 39, 40, 41), and *HACD1* mutations cause myopathy (42). A missense *TECR* mutation (P182L), which lowers the activity and stability of the protein product (43), leads to nonsyndromic mental retardation (44).

Mammalian TECR has been found to be involved in the fourth step of the FA elongation cycle based on sequence homology with yeast Tsc13 (17). Subsequently, we showed that TECR is also involved in the metabolism of the LCB sphingosine to glycerolipids (22, 45). In this sphingosine-to-glycerolipid metabolic pathway, C16:1 *trans*-2-enoyl-CoA (*trans*-2-C16:1-CoA) is generated as an intermediate, and TECR catalyzes its conversion to palmitoyl-CoA (C16:0-CoA). Analysis of yeast Tsc13 and *Arabidopsis* Tsc13 has shown that Tsc13 family proteins are integral membrane proteins containing six transmembrane domains, with both the N- and C-termini facing the cytosol (46).

To date, the catalytic mechanism of Tsc13/TECR and the amino acid residues important for their activity have remained largely unclear. Tsc13/TECR catalyze an NADPH-dependent reduction (saturation) of the carbon–carbon double bond between C2 and C3 of *trans*-2-enoyl-CoAs (17). A similar reaction is seen in bacterial and mitochondrial type II FA synthesis, which is catalyzed by *trans*-2-enoyl-acyl carrier protein (ACP) reductases (47, 48). Bacterial and mitochondrial *trans*-2-enoyl-ACP reductases belong to the short-chain dehydrogenase/reductase (SDR) and medium-chain dehydrogenase/reductase (MDR) superfamilies, respectively (49, 50, 51). In their reactions, a hydride ion and a proton are transferred to the C3 and C2 carbons from NADPH and the catalytic Tyr residue, respectively (47). Tsc13/TECR belong to neither SDR nor MDR and have no sequence motifs containing the Tyr catalytic residue that are conserved in SDR/MDR or bacterial/mitochondrial *trans*-2-enoyl-ACP reductases. Mutational analysis of Tsc13 has revealed that Lys140 and Arg141 are important for this activity (46), although their role in the catalysis is unknown. Therefore, the catalytic residues, especially Tyr (or other amino acid residues), that supply a proton to the C2 carbon of *trans*-2-enoyl-CoAs are unknown.

The enzymes responsible for the four reactions in the FA elongation cycle do not work independently but in concert by interacting with each other. For example, the presence of KAR and TECR are required for the full activity of ELOVLs and HACDs, respectively (43, 52). However, the cooperative action between TECR/Tsc13 and the FA elongases, which respectively catalyze the consecutive fourth reaction and first reaction of the next round of the FA elongation cycle, remains unclear. In the present study, through the analysis of yeast *TSC13* and human *TECR* mutants, we identified amino acid residues that are important for Tsc13/TECR activity, which include the catalytic Tyr residues, and for interaction with FA elongases. We also elucidated the cooperative action between Tsc13/TECR and FA elongases.

## Results

### Identification of amino acid residues important for Tsc13 activity

To obtain clues to the identity of the catalytic residues of Tsc13 family members, we compared the amino acid sequences of Tsc13 orthologs from 13 species (Fig. S1). We selected 15 of the highly conserved amino acid residues (Gly79, Glu91, Tyr92, Gly94, Lys140, Thr145, Thr155, Asn160, Lys163, Asn164, Cys165, Tyr168, Tyr179, His221, and Tyr256) and created their Ala-substituted mutants. Eleven of these (Glu91, Tyr92, Lys140, Thr145, Thr155, Lys163, Cys165, Tyr168, Tyr179, His221, and Tyr256) are capable of providing a proton. We did not select His137, Arg141, Glu144, His149, or Glu259 because their mutants have already been created and analyzed (46). We chose Gly79 and Gly94 because Gly residues are commonly found in NAD(P)H-binding motifs (53). The Asn residue is one of the catalytic tetrad (Asn/Ser/Tyr/Lys) of the SDR superfamily (49). Asn160 and Asn164 are located in the transmembrane helix (TH) 3 in the structural model obtained from the AlphaFold Protein Structure Database (ID Q99190) (54), where highly conserved amino acid residues are clustered.

The plasmid encoding WT *TSC13* or its respective *TSC13* mutants, each of which was tagged with *3×FLAG* at the 5′-terminus, were introduced into *tsc13*Δ cells expressing the mammalian ceramide synthase CERS5 (*tsc13*Δ/*CERS5*). Since yeast ceramide synthase (Lag1/Lac1/Lip1 complex), which exhibits substrate specificity toward VLC acyl-CoAs, cannot produce ceramides without VLCFA production (26), *tsc13*Δ cells do not survive (21). However, expression of the mammalian ceramide synthase CERS5, which is active toward C16:0-CoA, allows *tsc13*Δ cells to grow (45). Immunoblot analysis showed that expression levels of Tsc13 mutants were all comparable to that of WT protein (Fig. 1*B*).

We examined the growth of the cells under normal growth temperature (30 °C) or high temperature (38 °C). Although the cells expressing Tsc13 WT grew at 38 °C, those bearing vector showed temperature-sensitive growth (Fig. 1*C*). Expression of most of the Tsc13 mutant proteins allowed *tsc13*Δ/*CERS5* cells to grow at 38 °C similarly to cells expressing WT protein. However, cells expressing the E91A mutant grew only very weakly at 38 °C.

To examine the activity of each mutant, we performed an FA elongation assay by incubating membrane fractions with stearoyl-CoA (C18:0-CoA) and [^14^C]malonyl-CoA in the presence of NADPH. The acyl-CoAs and other FA elongation cycle intermediates produced were converted to FAs *via* alkaline hydrolysis and separated using TLC. The primary rate-limiting step in the FA elongation cycle is the first one (the condensation reaction), and the secondary rate-limiting step is the third one (the dehydration reaction) (43, 55). Therefore, the products yielded by the membrane fraction of WT Tsc13-expressing cells were acyl-CoAs (detected as FAs on TLC in this experimental system) and 3-OH acyl-CoAs (3-OH FAs on TLC), with the acyl-CoAs being the more abundant (Fig. 1*D*). Neither 3-ketoacyl-CoAs nor *trans*-2-enoyl-CoAs were detected. In contrast, vector-bearing cells did not produce acyl-CoA, but we instead detected *trans*-2-enoyl-CoA, a substrate of Tsc13. The G79A, G94A, T145A, N160A, K163A, and C165A mutants showed activity comparable to WT proteins, whereas the activity of the E91A, Y92A, K140A, T155A, N164A, Y168A, Y179A, H221A, and Y256A mutants was lower than that of WT. In the cells expressing Tsc13 with reduced activity, lower levels of acyl-CoAs and higher levels of *trans*-2-enoyl-CoAs were produced than in WT Tsc13-expressing cells. Particularly large decreases in activity were observed for cells expressing the Y168A or Y256A mutants. The Y256A mutant-expressing cells produced more *trans*-2-enoyl-CoAs than acyl-CoAs, and the Y168A mutant-expressing cells produced similar quantities of acyl-CoAs and *trans*-2-enoyl-CoAs. These results indicate that the Tyr168 and Tyr256 residues are especially important for this activity. An inverse correlation between the quantities of acyl-CoAs and *trans*-2-enoyl-CoAs was observed for most mutants. However, the E91A mutant did not produce the levels of acyl-CoAs expected based on those of *trans*-2-enoyl-CoAs. Indeed, the quantities of *trans*-2-enoyl-CoAs in the E91A mutant-expressing cells were comparable to those in the N164A or Y179A mutant-expressing cells, but their levels of acyl-CoAs were much lower. As a result, the total quantities of acyl-CoAs and other FA elongation cycle intermediates (3-OH acyl-CoAs and *trans*-2-enoyl-CoAs) were lower in the E91A mutant-expressing cells than in the N164A or Y179A mutant-expressing cells (as well as in all other mutant and WT Tsc13-expressing cells). This result suggests that in the E91A mutant-expressing cells, not only the fourth step (reduction) of the FA elongation cycle is impaired but that the first, rate-limiting step (condensation) of the cycle may also be affected.

In yeast, acyl-CoAs are elongated to C26-CoA *via* multiple rounds of FA elongation cycles. To examine the chain length of acyl-CoAs elongated in *tsc13*Δ/*CERS5* cells expressing Tsc13 mutants, we then performed an FA elongation assay using C18:0-CoA and stable isotope-labeled [^13^C]malonyl-CoA, followed by quantification *via* LC coupled with tandem mass spectrometry (MS/MS). In vector-bearing *tsc13*Δ/*CERS5* cells, little C20:0 acyl-CoA (C20:0-CoA) was produced, but a substantial quantity of *trans*-2-C20:1-CoA was detected (Fig. 1, *E* and *F*). In contrast, in WT Tsc13 protein-expressing cells, C20:0-CoA, C22:0-CoA, C24:0-CoA, and C26:0-CoA were produced, with almost no *trans*-2-enoyl-CoA intermediates. In cells expressing the Y256A mutant, whose activity was found to be greatly reduced in the above assay (Fig. 1*D*), the production of acyl-CoAs, irrespective of the chain length, was reduced compared to WT protein-expressing cells, and instead *trans*-2-C20:1-CoA and *trans*-2-C22:1-CoA were detected (Fig. 1, *E* and *F*). The E91A mutant-expressing cells produced similar quantities of *trans*-2-C20:1-CoA to the Y256A mutant-expressing cells, but the quantity of C20:0-CoA they produced was much lower. This result was consistent with the above hypothesis that condensation activity was reduced in the E91A mutant-expressing cells, in which ≥C22:0 acyl-CoAs were almost absent. In summary, we found that nine amino acid residues (Glu91, Tyr92, Lys140, Thr155, Asn164, Tyr168, Tyr179, His221, Tyr256), especially Glu91 and Tyr256, are important for Tsc13 activity.

### Large decrease in the activity of the E91A mutant *in vivo*

**Figure 2:**
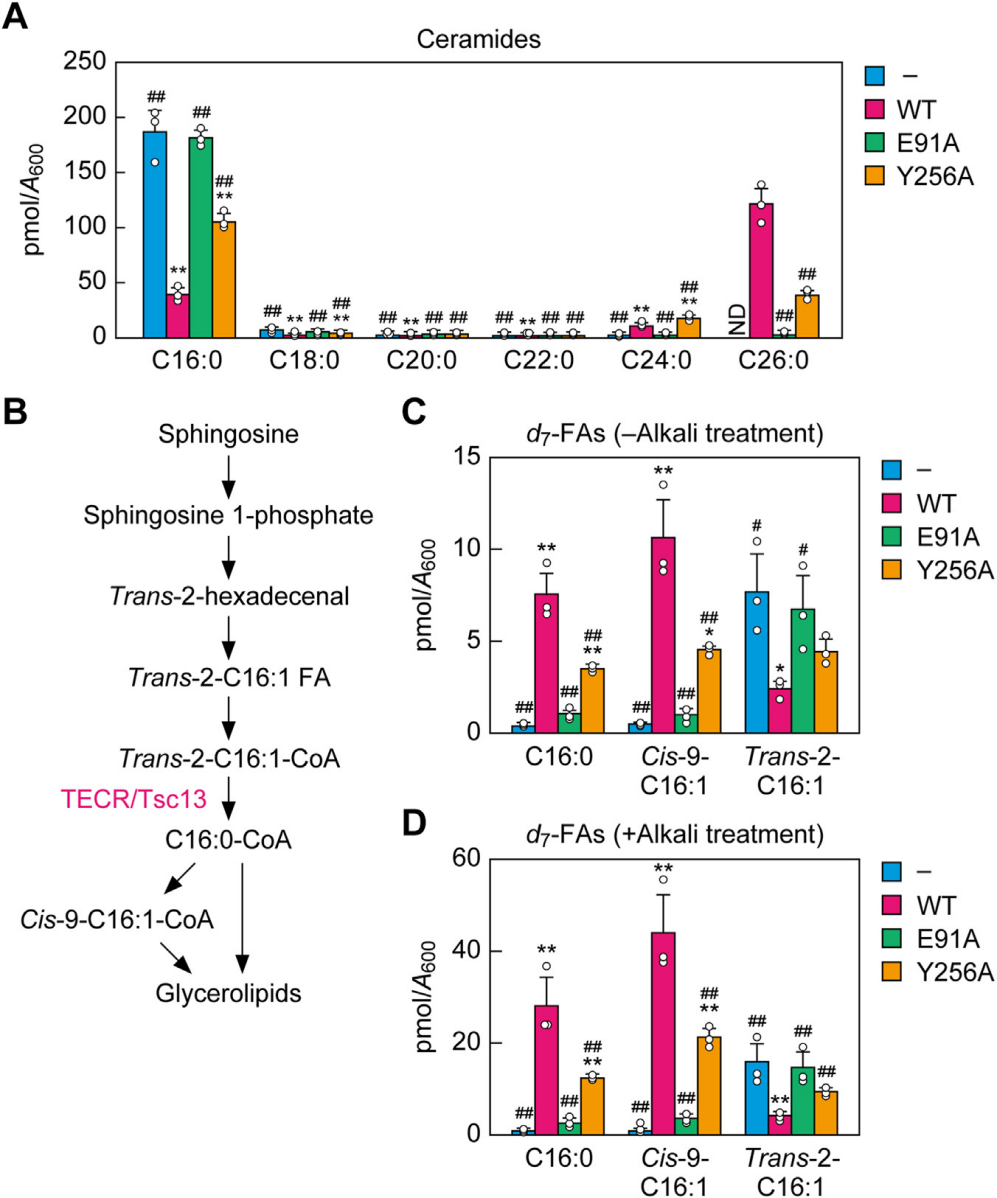
**Effects of *E91A* and *Y256A* mutations on Tsc13 activity *in vivo*.***A*, *C*, and *D*, ABY80 (*tsc13*Δ/*CERS5*) cells harboring the vector or the plasmid encoding *3×FLAG-TSC13* (WT, *E91A*, or *Y256A*) were grown to early log-phase at 30 °C. *A*, lipids were extracted from the cells, and ceramides containing a phytosphingosine and a 2-OH FA were quantified *via* LC-MS/MS. Values are the quantities of ceramides with the respective chain length/degree of unsaturation of the 2-OH FA moiety and are the means + SD from three independent experiments. Statistically significant differences are indicated (Dunnett’s test; ∗*p* < 0.05, ∗∗*p* < 0.01 [*versus* vector control]; ^#^*p* < 0.05; ^##^*p* < 0.01 [*versus* WT]). *B*, metabolic pathway of sphingosine to glycerolipids. Sphingosine is metabolized to C16:0-CoA *via* sphingosine 1-phosphate, *trans*-2-hexadecenal, *trans*-2-hexadecenoic acid (*trans*-2-C16:1 FA), and *trans*-2-hexadecenoyl-CoA (*trans*-2-C16:1-CoA). The C16:0-CoA generated is incorporated into glycerolipids directly or after desaturation to *cis*-9-C16:1-CoA. *C* and *D*, cells were labeled with 1 μM *d*_7_-sphingosine at 30 °C for 30 min. Lipids were extracted from cells and either directly (*C*) or following alkali treatment (*D*) subjected to FA derivatization with AMP amide and quantification of *d*_7_-labeled FAs *via* LC-MS/MS. Values are the means + SD from three independent experiments. Statistically significant differences are indicated (Dunnett’s test; ∗∗*p* < 0.01 [*versus* vector control]; ^#^*p* < 0.05; ^##^*p* < 0.01 [*versus* WT]). ND, not detected.

We next examined the FA elongation activity in *tsc13*Δ/*CERS5* cells expressing the E91A or Y256A mutant *in vivo*. Since most of the VLCFAs produced are converted to ceramides/sphingolipids in yeast, we quantified the ceramides composed of a phytosphingosine and a 2-OH FA (the most abundant ceramide class in yeast) *via* LC-MS/MS. In vector-bearing *tsc13*Δ/*CERS5* cells, almost all (∼95%) of the ceramide species had the C16:0 FA moiety (C16:0 ceramide), which is produced by ectopically expressed CERS5 (Fig. 2*A*). However, C26:0 ceramide (produced by yeast ceramide synthase) was the most abundant ceramide in WT Tsc13-expressing cells, followed by C16:0 ceramide. The ceramide composition in E91A mutant-expressing cells was similar to that in vector-bearing cells. The Y256A mutant-expressing cells showed a ceramide composition that was intermediate between vector-bearing cells and WT Tsc13-expressing cells: their levels of C26:0 and C24:0 ceramides were lower than WT Tsc13-expressing cells, and their levels of C16:0 ceramide were lower than in vector-bearing cells.

The above method revealed the *in vivo* activity of the FA elongation cycle as a whole rather than that of Tsc13 alone. To investigate the latter, we next performed a *d*_7_ (seven deuterium)-sphingosine labeling assay. In mammals, sphingosine is metabolized not only to ceramides/sphingolipids but also to glycerolipids. In the latter pathway, sphingosine with C18 is metabolized to C16:0-CoA *via* five reactions (Fig. 2*B*) (22, 56). Of these, the last one—the conversion of *trans*-2-C16:1-CoA to C16:0-CoA—is catalyzed by TECR (45). The C16:0-CoA produced is then incorporated into glycerolipids directly or after desaturation to palmitoleoyl-CoA (*cis*-9-C16:1-CoA) (57). Sphingosine is not an LCB that occurs naturally in yeast. However, when it is added to yeast exogenously, it is metabolized to glycerolipids *via* a similar process to the one in mammals (56). Further, Tsc13 catalyzes the conversion of *trans*-2-C16:1-CoA to C16:0-CoA (45). In the *d*_7_-sphingosine labeling assay, lipids were extracted from the cells and either not treated with anything (to allow measurement of free *d*_7_-labeled FAs) or treated with alkali (for measurement of *d*_7_-labeled FAs incorporated into ester-bound lipids [mainly glycerolipids] in addition to free *d*_7_-labeled FAs), followed by quantification *via* LC-MS/MS. In the measurements of free *d*_7_-labeled FAs, substantial quantities of C16:0 FA and the derived *cis*-9-C16:1 FA were detected in *tsc13*Δ/*CERS5* cells expressing WT Tsc13 (Fig. 2*C*). A small quantity of *trans*-2-C16:1 FA, which had the *trans* double bond that was originally in the sphingosine, was detected. In vector-bearing cells, little C16:0 FA or *cis*-9-C16:1 FA were produced, and *trans*-2-C16:1 FA accumulated instead. The E91A mutant-expressing cells showed a similar FA production pattern to vector-bearing cells. In the Y256A mutant-expressing cells, C16:0 FA and *cis*-9-C16:1 FA levels were 48% and 43% of those in the WT Tsc13-expressing cells, respectively. Similar results were obtained for the alkali-treated samples, although alkali treatment increased the quantities of *d*_7_-labeled FAs approximately fourfold relative to no treatment (Fig. 2*D*). These results indicate that the *in vivo* activity of both of these mutants was reduced, although the decrease was more pronounced in the E91A mutant.

### Loss of activity of the double mutants of the three Tyr residues

**Figure 3:**
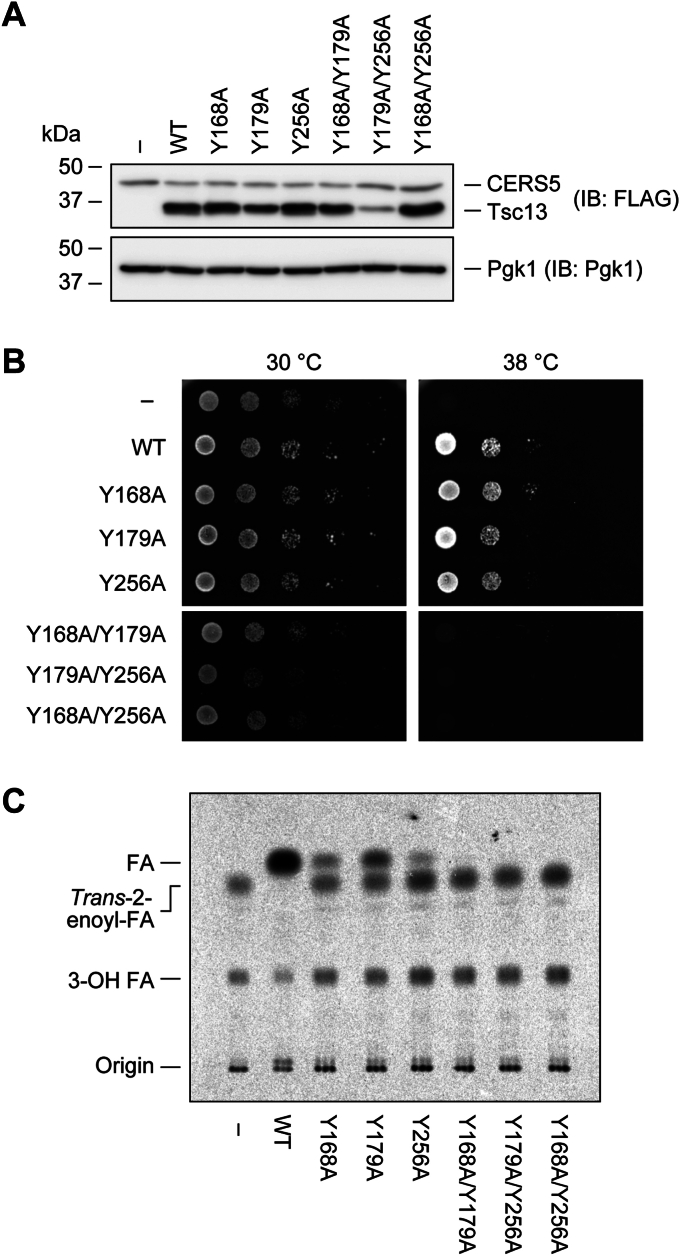
**Effect of double mutations of Tyr residues on Tsc13 activity.** ABY80 (*tsc13*Δ/*CERS5*) cells harboring the vector or the plasmid encoding *3×FLAG-TSC13* (WT or each mutant) were grown to early log-phase at 30 °C. *A*, total cell lysates were prepared from the cells and separated by SDS-PAGE, followed by immunoblotting with anti-FLAG and anti-Pgk1 (loading control) antibodies. *B*, cells were diluted to *A*_600_ = 1.5 and further serially diluted 10-fold. Cells were spotted onto plates of SC–His–Ura and incubated at 30 °C or 38 °C. *C*, membrane fractions were prepared and subjected to an *in vitro* FA elongation assay. Membrane fractions (10 μg) were incubated with 20 μM C18:0-CoA, 27.3 μM [^14^C]malonylCoA, and 1 mM NADPH at 37 °C for 30 min. Acyl-CoAs were alkaline-hydrolyzed to FAs, neutralized, extracted, separated by TLC, and detected using a bioimaging analyzer BAS-2500. IB, immunoblotting.

The activity of the Y168A, Y179A, and Y256A mutants was lower than that of WT Tsc13, and in decreasing order of activity, they were Y256A, Y168A, and Y179A (Fig. 1*D*). To obtain a clue as to the roles of these Tyr residues, we created double mutants (Y168A/Y179A, Y179A/Y256A, and Y168A/Y256A). The expression levels of these mutant proteins in the *tsc13*Δ/*CERS5* cells were comparable to those of the WT protein, except for the Y179A/Y256A double mutant, which showed lower levels (Fig. 3*A*). In contrast to their respective single mutants, neither of these double mutant proteins restored the temperature sensitivity of *tsc13*Δ/*CERS5* cells (Fig. 3*B*). An *in vitro* FA elongation assay showed that all of these double mutants completely lost activity (Fig. 3*C*). These results indicate that these three Tyr residues are important for catalysis or structure formation (see Discussion).

### Reduced condensation activity in cells expressing the E91A Tsc13 mutant

**Figure 4:**
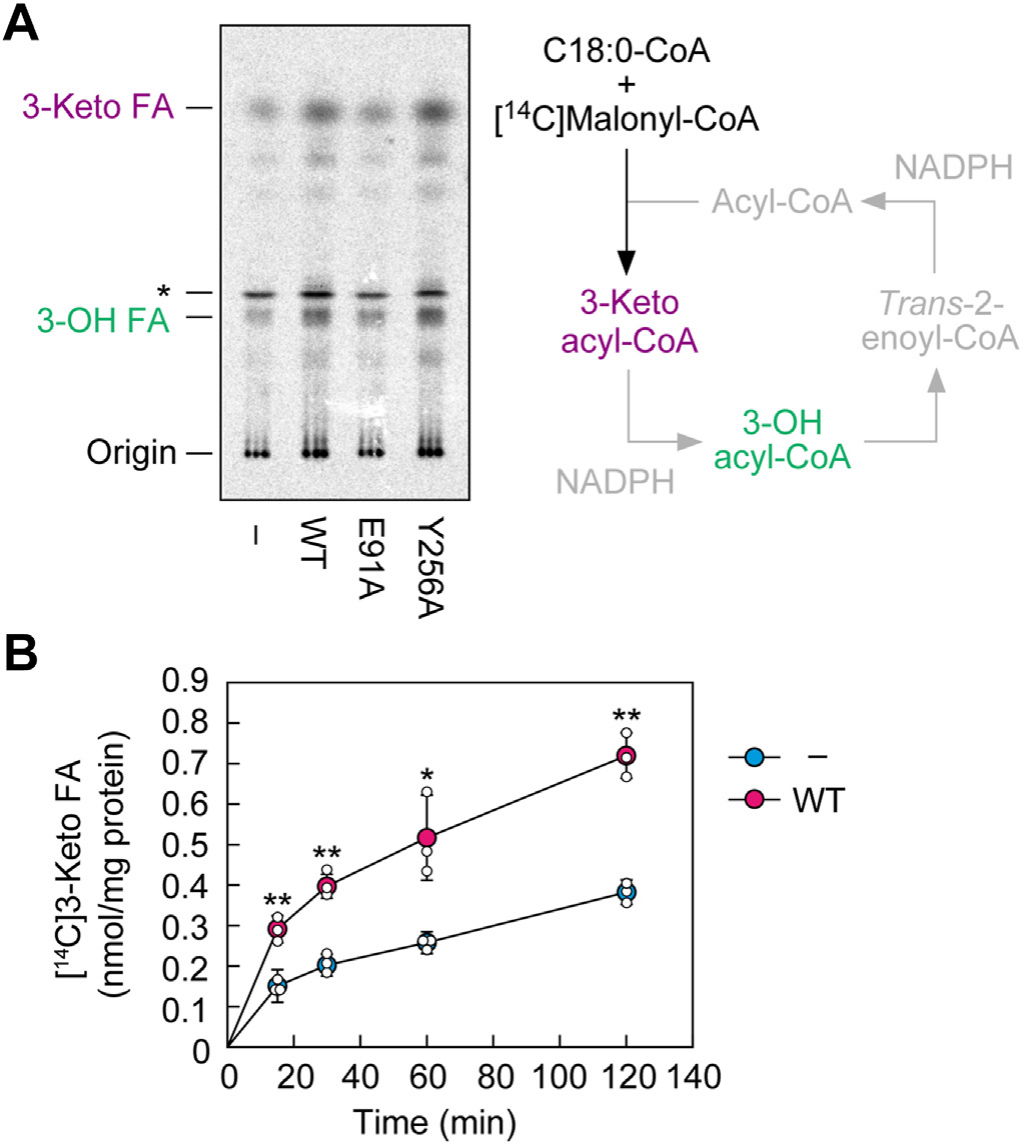
**Reduced condensation activity due to *TSC13 E91A* mutation.***A* and *B*, membrane fractions were prepared from ABY80 (*tsc13*Δ/*CERS5*) cells harboring the vector (*A* and *B*) or the plasmid encoding *3×FLAG-TSC13* (WT, *A* and *B*; *E91A* or *Y256A*, *A*). The membrane fractions were incubated with 20 μM C18:0-CoA and 27.3 μM [^14^C]malonylCoA in the absence of NADPH at 37 °C for 30 min (*A*) or for 15, 30, 60, and 120 min (*B*). Acyl-CoAs were alkaline-hydrolyzed to FAs, neutralized, extracted, separated *via* TLC, and detected and quantified using a bioimaging analyzer BAS-2500. The asterisk indicates an unidentified lipid. The schematic diagram of the assay system is shown on the *right* (*A*). Reactions in *gray* do not proceed due to the absence of NADPH. Values are means ± SD from three independent experiments (*B*). Statistically significant differences are indicated (Student’s *t* test, unpaired; ∗*p* < 0.05, ∗∗*p* < 0.01).

The results of the FA elongation assay raised the possibility that the activity of the first, rate-limiting step (condensation reaction) of the FA elongation cycle was reduced in *tsc13*Δ*/CERS5* cells expressing the E91A mutant (Fig. 1, *D*–*F*). To examine this possibility, we performed an FA elongation assay in the absence of NADPH. Since the second step of the FA elongation cycle (reduction) requires NADPH as a cofactor, only condensation activity could be evaluated in the assay without NADPH. Compared to vector-bearing *tsc13*Δ/*CERS5* cells, cells expressing WT Tsc13 produced greater quantities of 3-ketoacyl-CoAs (Fig. 4*A*), indicating that the activity of FA elongases catalyzing the condensation reaction was higher in the presence of Tsc13 than in its absence. In this assay, 3-OH acyl-CoAs were also produced, probably due to inclusion of endogenous NADPH in the membrane fractions used. The quantity of 3-ketoacyl-CoAs produced in cells expressing the Y256A mutant, which was used as a control with reduced activity, was comparable to that in WT protein-expressing cells. This result suggests that a decrease in Tsc13 activity does not affect condensation activity. However, the levels of 3-ketoacyl-CoAs produced in the E91A mutant-expressing cells were as low as those of vector-bearing cells, indicating that their condensation activity was reduced. One possible reason for this is that the interaction between the Tsc13 and the FA elongases Elo2/Elo3, which catalyze the condensation reaction, was impaired in the E91A mutant.

In the above experiment, the reaction time was fixed at 30 min, leaving open the possibility that the production of 3-ketoacyl-CoAs had already plateaued by this point. We therefore repeated the assay with reaction time points at 15, 30, 60, and 120 min to examine the reaction rates of FA elongases in the presence and absence of Tsc13. At each time point, the activity of the FA elongases in the presence of Tsc13 was approximately double that in its absence (Fig. 4*B*).

### Reduced interaction between Tsc13 E91A mutant and Elo2/Elo3

**Figure 5:**
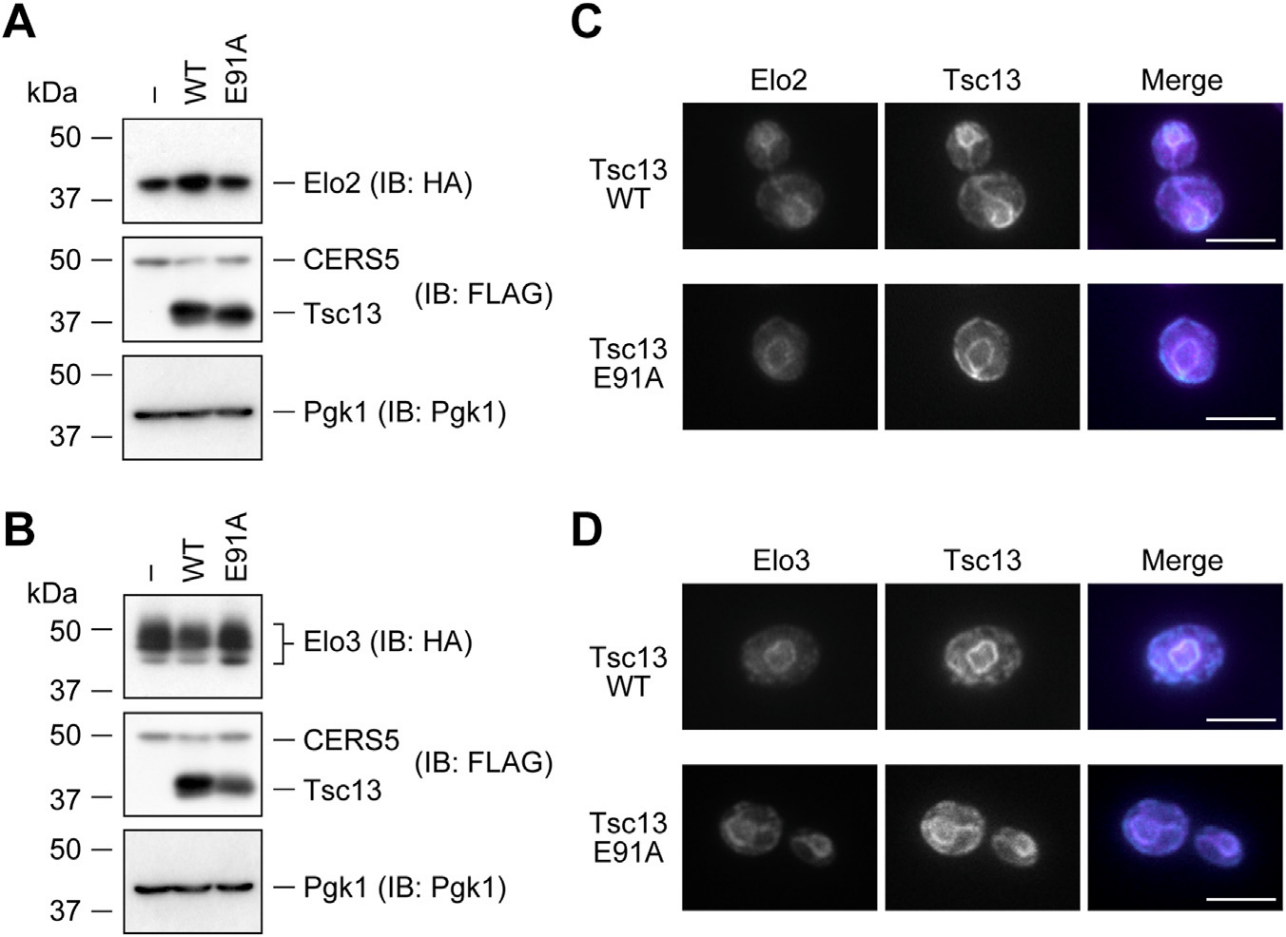
**Effect of Tsc13 E91A mutant on the cellular quantities and localization of Elo2 and Elo3.** YTY8 (*tsc13*Δ *ELO2-6×HA*/*CERS5*; *A* and *C*) and YTY9 (*tsc13*Δ *ELO3-6×HA*/*CERS5*; *B* and *D*) cells, each harboring the vector or the plasmid encoding *3×FLAG-TSC13* (WT, *E91A*, or *Y256A*), were grown to early log-phase at 30 °C. *A* and *B*, total cell lysates were prepared from the cells and separated *via* SDS-PAGE, followed by immunoblotting with anti-HA, anti-FLAG, and anti-Pgk1 (loading control) antibodies. *C* and *D*, cells were subjected to indirect immunofluorescence microscopy using anti-FLAG (*magenta*) and anti-HA (*cyan*) antibodies. Scale bars represent 5 μm. IB, immunoblotting.

To reveal the mechanism of the reduced condensation activity in the Tsc13 E91A mutant-expressing cells, we created yeast strains in which a *6×HA* tag was chromosomally fused to the 3′-terminus of the *ELO2* or *ELO3* gene in the *tsc13*Δ/*CERS5* cells (*tsc13*Δ *ELO2-6×HA*/*CERS5* or *tsc13*Δ *ELO3-6×HA*/*CERS5* cells, respectively). We then introduced a plasmid encoding WT *TSC13* or *TSC13 E91A*, each tagged with *3×FLAG*, or an empty vector into these cells. Immunoblot analysis showed that the expression levels of both Elo2 and Elo3 were comparable among vector-bearing cells, WT Tsc13-expressing cells, and E91A mutant-expressing cells (Fig. 5, *A* and *B*). These results indicate that the reduced condensation activity in the vector-bearing or E91A mutant-expressing cells was not due to reduced levels of Elo2 or Elo3.

Proteins involved in the FA elongation cycle, including Tsc13, are all localized in the ER (46). We used indirect immunofluorescence microscopy to examine whether expression of the E91A mutant affected the subcellular localization of Elo2 and Elo3. They showed a double ring structure, which is typical of yeast ER (since it consists of nuclear and cortical ER), in both the WT Tsc13- and the E91A mutant-expressing cells (Fig. 5, *C* and *D*). WT Tsc13 and E91A mutant proteins showed similar localization patterns and were colocalized with Elo2 and Elo3. Thus, the reduced condensation activity in E91A mutant-expressing cells was not due to abnormal localization of Elo2 or Elo3.

**Figure 6:**
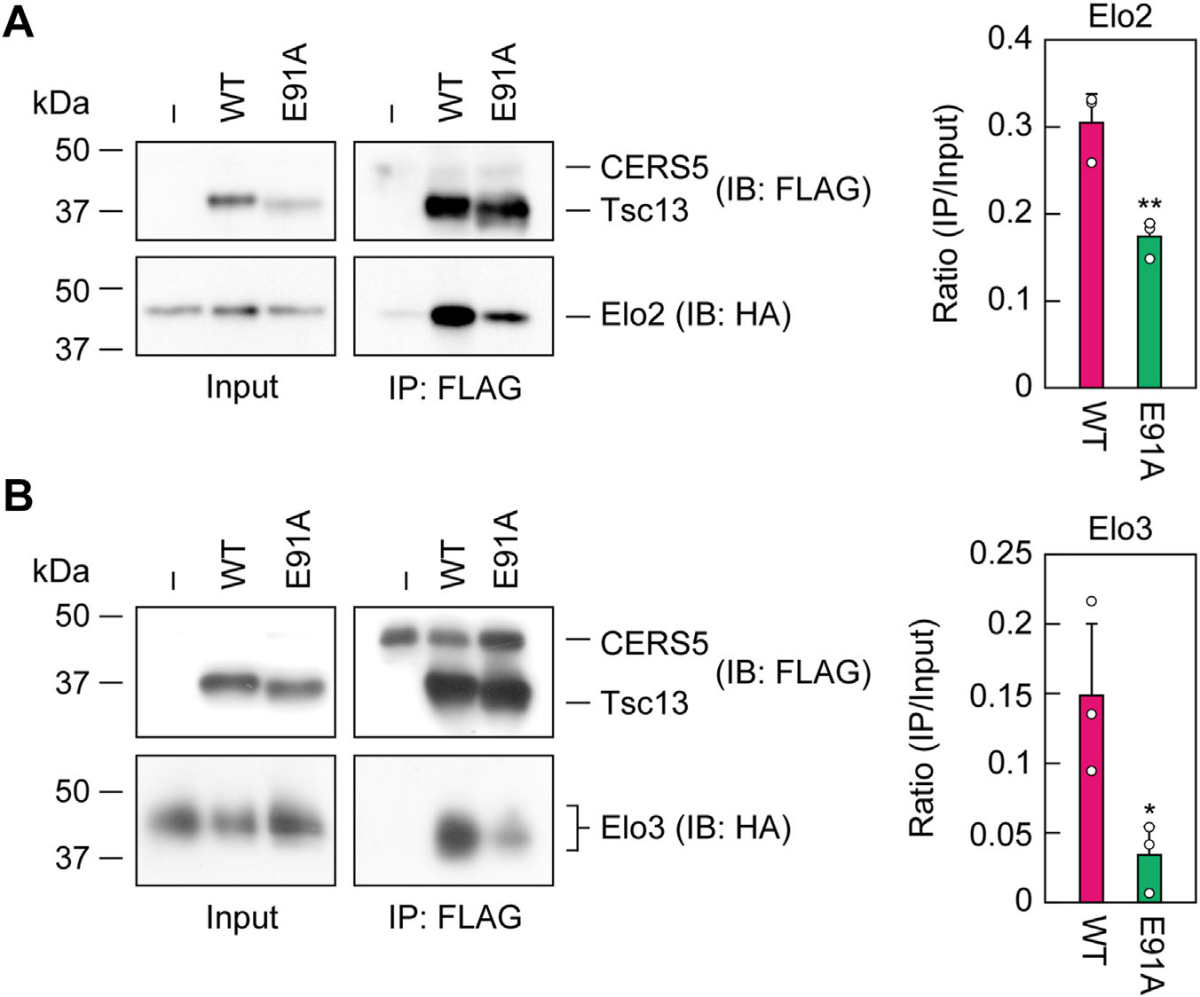
**Reduced interaction between Tsc13 and Elo2/Elo3 by *E91A* mutation.** YTY8 (*tsc13*Δ *ELO2-6×HA*/*CERS5*; *A*) and YTY9 (*tsc13*Δ *ELO3-6×HA*/*CERS5*; *B*) cells, each harboring the vector or the plasmid encoding *3×FLAG-TSC13* (WT or *E91A* mutant), were grown to early log-phase at 30 °C. Membrane fractions prepared from the cells were solubilized with 1% Triton X-100 and subjected to immunoprecipitation with anti-FLAG antibody. Solubilized proteins (input; *left* panels) and immunoprecipitated proteins (*right* panels; 12 × the quantities of the solubilized proteins) were subjected to immunoblotting with anti-FLAG and anti-HA antibodies. The graphs show the ratios of the band intensity in the immunoprecipitation fraction relative to the input. Values are means + SD from three independent experiments. Statistically significant differences are indicated (Student’s *t* test, unpaired; ∗*p* < 0.05, ∗∗*p* < 0.01). IB, immunoblotting; IP, immunoprecipitation.

We next examined the interaction between Tsc13 and Elo2/Elo3 *via* co-immunoprecipitation. The membrane fraction of *tsc13*Δ *ELO2-6×HA*/*CERS5* cells expressing WT 3×FLAG-Tsc13 were solubilized with Triton X-100 and subjected to immunoprecipitation using anti-FLAG antibody. In the immunoprecipitated fraction, Elo2-6×HA was detected together with 3×FLAG-Tsc13, indicating that Tsc13 and Elo2 interact (Fig. 6*A*). However, the quantity of Elo2-6×HA detected in the immunoprecipitated fraction from E91A mutant-expressing cells was reduced to about 57% of that in WT Tsc13-expressing cells. A similar result was obtained for Elo3: the quantity of Elo3-6×HA in the immunoprecipitation fraction from the mutant-expressing cells was reduced to 21% of that in the same fraction from WT Tsc13-expressing cells (Fig. 6*B*). Combined, these results indicate that the reduced condensation activity in E91A mutant-expressing cells was due to reduced interaction between Tsc13 and Elo2/Elo3.

### Conservation of the roles of amino acid residues between yeast Tsc13 and human TECR

**Figure 7:**
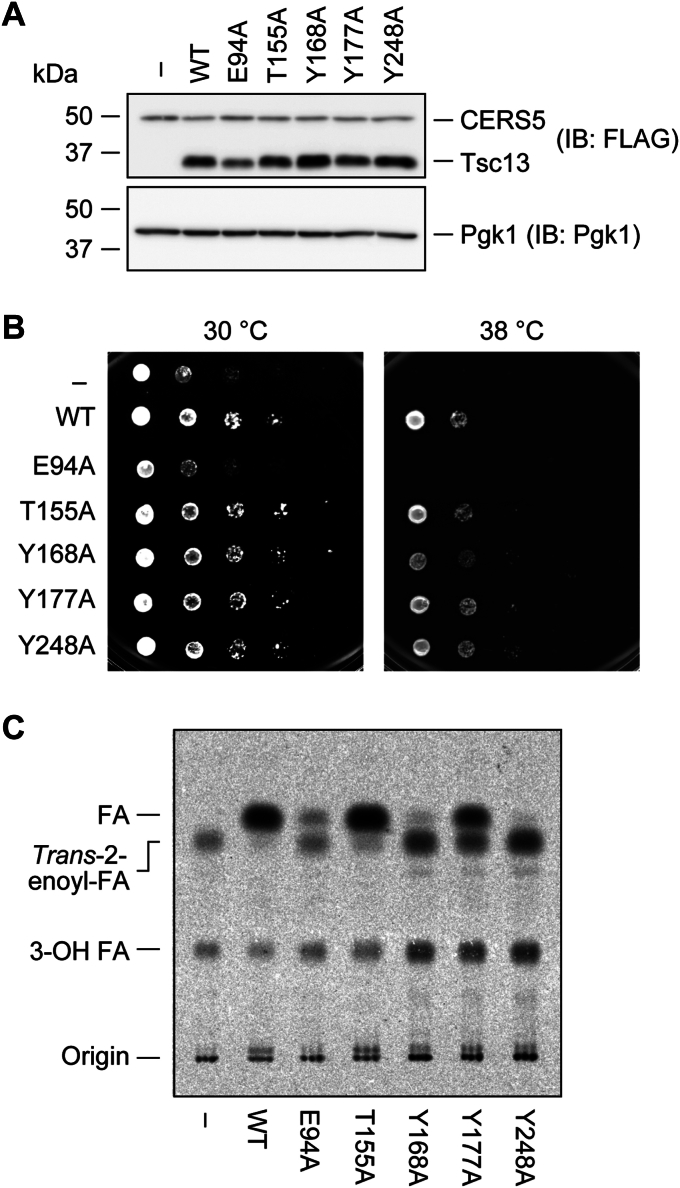
**Importance of Tyr168 and Tyr248 residues in the activity of TECR.** ABY80 (*tsc13*Δ/*CERS5*) cells harboring the vector or the plasmid encoding *3×FLAG-TECR* (WT or each mutant) were grown to early-log phase at 30 °C. *A*, total cell lysates were prepared from the cells and separated *via* SDS-PAGE, followed by immunoblotting with anti-FLAG and anti-Pgk1 (loading control) antibodies. *B*, cells were diluted to *A*_600_ = 1.5 and further serially diluted 10-fold. Cells were spotted onto plates of SC –His –Ura and incubated at 30 °C or 38 °C. *C*, membrane fractions were prepared and subjected to an *in vitro* FA elongation assay. Membrane fractions (10 μg) were incubated with 20 μM C18:0-CoA, 27.3 μM [^14^C]malonylCoA, and 1 mM NADPH at 37 °C for 30 min. Acyl-CoAs were alkaline-hydrolyzed to FAs, neutralized, extracted, separated *via* TLC, and detected using a bioimaging analyzer BAS-2500. IB, immunoblotting.

To examine whether the roles of the amino acid residues of Tsc13 are conserved in human TECR, Ala substitution mutants of Glu94, Thr155, Tyr168, Tyr177, and Tyr248 in TECR, corresponding to Glu91, Thr155, Tyr168, Tyr179, and Tyr256, respectively, in Tsc13, were created (Table 1). When these mutant TECR proteins were expressed in the *tsc13*Δ/*CERS5* cells, their expression levels were comparable to those of WT TECR protein (Fig. 7*A*). Next, we examined the growth of the cells expressing WT TECR or the mutants at 30 °C and 38 °C and found that only those expressing the E94A mutant were sensitive to temperature (Fig. 7*B*), which was similar to the case of the corresponding yeast Tsc13 E91A mutant (Fig. 1*C*). Finally, we conducted an FA elongation assay by incubating membrane fractions of cells expressing WT TECR or the mutants with [^14^C]malonyl-CoA and C18:0-CoA in the presence of NADPH. WT protein-expressing cells produced [^14^C]acyl-CoAs, and no *trans*-2-enoyl-CoA intermediates were detected (Fig. 7*C*). The T155A mutant-expressing cells produced similar levels of acyl-CoAs to WT protein-expressing cells, but a small quantity of *trans*-2-enoyl-CoAs was detected, indicating that the T155A mutant was slightly less active than the WT protein. The Y177A, Y168A, and Y248A mutants were much less active, in this order, with the Y248A mutant exhibiting almost no activity. In the E94A mutant-expressing cells, low levels of acyl-CoAs were detected, along with the similar levels of *trans*-2-enoyl-CoAs. The total quantities of FA elongation cycle intermediates (acyl-CoAs + *trans*-2-enoyl-CoAs + 3-OH acyl-CoAs) detected in E94A mutant-expressing cells were lower than those in WT- or other mutant-expressing cells. This result was similar to that observed for the yeast Tsc13 E91A mutant (Fig. 1*D*). Thus, the roles of the amino acid residues we have revealed for Tsc13 are likely to be conserved in TECR.

**Table 1:** The yeast Tsc13 mutants and their corresponding human TECR mutants created in this study

Tsc13	TECR
E91A	E94A
T155A	T155A
Y168A	Y168A
Y179A	Y177A
Y256A	Y248A

## Discussion

**Figure 8:**
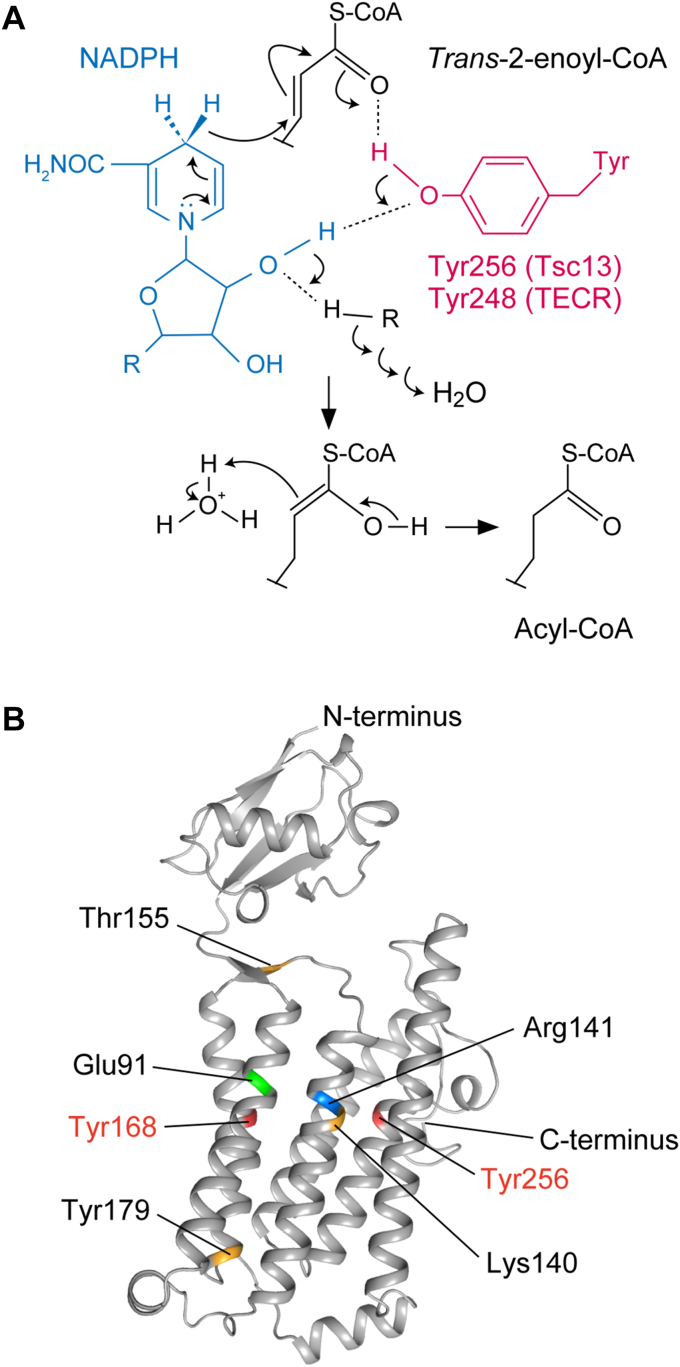
**Models of catalytic mechanism and three-dimensional structureof Tsc13.***A*, catalytic mechanism model for the reduction of *trans*-2-enoyl-CoA by Tsc13. A hydride ion is transferred from NADPH to the C3 of the double bond of *trans*-2-enoyl-CoA, producing an enolate anion on the C1 carbonyl oxygen. Tyr256 of Tsc13 (Tyr248 of TECR) donates a proton to the enolate anion. The resulting enol is tautomerized to acyl-CoA. The proton of Tyr256 is replenished *via* a proton relay system through the 2′-OH of the ribose portion of NADPH, several Tsc13 amino acid residues (Tyr168, Lys140, and/or Arg141), and water. *B*, the structural model of Tsc13 obtained from the AlphaFold Protein Structure Database is shown. Some amino acid residues whose activity was found to be reduced by Ala-substitution in this study were mapped on the structure using the PyMOL software and together with those identified in a previous report (46) are marked in color.

The catalytic residues of Tsc13/TECR have until now been unknown. The production of FAs by FASs occurs *via* four reactions (condensation, reduction, dehydration, and reduction), as in the FA elongation cycle, although the FAs are bound to ACP rather than CoA in the FASs-catalyzed reactions. Mammalian type I FASs in the cytosol are multifunctional enzymes, and they catalyze all of the above four reactions (58). In contrast, in mitochondrial and bacterial type II FA synthesis, each reaction is catalyzed by a different enzyme (47, 48). Bacterial *trans*-2-enoyl-ACP reductases belong to the SDR superfamily. The SDRs are NAD(P)H-dependent oxidoreductases involved in the metabolism of lipids, amino acids, carbohydrates, coenzymes, and hormones (53). In SDRs, the catalytic tetrad, Asn/Ser/Tyr/Lys, is commonly present (49). In the model proposed for the reduction of keto groups by 3β/17β-hydroxysteroid dehydrogenase, a hydride ion and a proton are supplied by NADH and Tyr151, respectively, and a proton relay occurs from water bound to Asn111, to Lys155, to the 2′-OH of the ribose moiety of NADH, to Tyr151 (59). The classic type of active center motif of SDRs is considered to be YxxxK, although YxxMxxxK and YxxxN have also been reported (53). The catalytic residues of the *trans*-2-enoyl-ACP reductase FabI in *E. coli* are Tyr156, Lys163, and Ser120 (47). A reaction mechanism model for this *trans*-2-enoyl-ACP reductase, which is similar to that for 3β/17β-hydroxysteroid dehydrogenase, has been proposed as follows (47, 60): A hydride ion is transferred from NADPH to the C3 of the C2–C3 double bond of *trans*-2-enoyl-ACP to form an enolate anion on the C1 carbonyl oxygen, which receives a proton from Tyr156. The resulting enol undergoes tautomerization to produce acyl-ACP. In this case, a proton relay occurs from water bound to Ser120, to Lys163, to the 2′-OH of the ribose moiety of NADPH, to Tyr156. Mitochondrial *trans*-2-enoyl-ACP reductases belong to the MDR superfamily (50). The MDR superfamily includes NAD(P)H-dependent enzymes involved in the oxidation/reduction of carbon–carbon double bonds or hydroxyl/keto groups such as alcohol dehydrogenases and leukotriene B_4_ dehydrogenases (51). Three-dimensional structural analyses of MECR and Etr1, the *trans*-2-enoyl-ACP reductases in human and *Candida tropicalis*, respectively, have shown that the catalytic residue is again Tyr (MECR, Tyr94; Etr1, Tyr79) (50, 61). Although the *trans*-2-enoyl-CoA reductases Tsc13/TECR catalyze a reaction similar to the one catalyzed by *trans*-2-enoyl-ACP reductases in bacterial and mitochondrial type II FA synthesis, they belong neither to the SDR nor the MDR superfamily. Therefore, we could not predict which Tyr residues (or indeed other amino acid residues) of Tsc13/TECR would act as catalytic residues based on sequence homology to SDR/MDR. In the present study, mutant analyses revealed that Tyr256/Tyr248 and Tyr168/Tyr168 in Tsc13/TECR are important for activity (Figs. 1 and 7). Of these Tyr-residue mutants, the mutant of Tyr248 of TECR (Y248A) showed almost no activity (Fig. 7*C*), suggesting that Tyr248 of TECR and its corresponding Tyr256 in Tsc13 act as catalytic residues. We thus propose a model for the catalytic mechanism of Tsc13 based on that for FabI (47, 60): Tyr256 (Tyr248 in TECR) provides a proton to the C2 of the *trans*-2-enoyl-CoAs through protonation of the enolate anion on the C1 carbonyl oxygen, which is generated by a hydride ion transfer from NADPH and subsequent tautomerization to a keto (Fig. 8*A*). In this reaction, the 2′-OH of the ribose moiety of NADPH acts as part of the proton relay. Although the three-dimensional structure of Tsc13 has not yet been revealed, a model for it has been proposed in the AlphaFold Protein Structure Database (https://alphafold.ebi.ac.uk; UniProt ID Q99190) (Fig. 8*B*). In this model, Tyr168 and Tyr256 are located close to each other. It is possible that Tyr168 is involved in part of the proton relay. The Y256A mutant showed residual activity (Fig. 1), while the Y168A/Y256A double mutant displayed no activity (Fig. 3). Considering these results, we speculate that in the Y256A mutant, where Tyr256 cannot act as the catalytic residue, Tyr168 fulfills that function instead. Lys140 and Arg141 were shown to be important for activity in a previous study (46), and the present study has confirmed this for Lys140. These residues are also in close proximity to the Tyr residues in the three-dimensional structure model (Fig. 8*B*). Therefore, the Lys140 and Arg141 residues may also be involved in the catalysis as parts of the proton relay or through binding to water, which acts as the proton source.

The Y179A mutant of Tsc13 showed reduced activity relative to WT protein (Fig. 1), and the Y168A/Y179A and Y179A/Y256A double mutants displayed no activity (Fig. 3). The three-dimensional structure model predicts that Tyr179 is located away from the active center, at the interface between the TH and the ER lumen (Fig. 8*B*). Therefore, we speculate that Tyr179 is not directly involved in the catalysis, but rather functions to maintain the structure. The cellular levels of the Y179A/Y256A double mutant were low (Fig. 3*A*), suggesting that the structural change may have caused protein destabilization.

We then found that Tsc13 and Elo2/Elo3 interact and that the activity of Elo2/Elo3 is reduced in cells without Tsc13 (vector-bearing *tsc13*Δ/*CERS5* cells) (Fig. 4). It is highly likely that the absence of the complex-forming partner caused the structure of Elo2/Elo3 to be impaired, resulting in reduced activity. We found similarly reduced Elo2/Elo3 activity in Tsc13 E91A mutant-expressing cells (Fig. 4*A*) and concluded that this was attributable to reduced interaction between the E91A mutant and Elo2/Elo3 (Fig. 6). Glu91 is located near the active center in the structural model of Tsc13 (Fig. 8*B*), suggesting that the active center is located near the interface between Tsc13 and Elo2/Elo3. The E91A mutant showed weak activity (Fig. 2). One possible reason for this is that Glu91 is directly involved in the catalysis. Another possibility, which we think more likely, is that the reduced interaction between Tsc13 and Elo2/Elo3 resulting from the *E91A* mutation impairs proper structure formation of not only Elo2/Elo3 but also Tsc13.

*Tecr* KO mice are embryonic lethal and do not survive beyond embryonic day 10.0 (31), and endothelial cell-specific *Tecr* KO mice show impaired angiogenesis involving delayed vascular sprouting (62). In humans, a missense mutation of *P182L* in *TECR* causes mental retardation (44). The *P182L* mutation is relatively weak: it causes only ∼30% reductions in C24:1 ceramide and sphingomyelin levels (43). This suggests that the nervous system is the most sensitive of the tissues to a decrease in VLCFA levels. Thus, TECR is physiologically and pathologically important.

In the present study, we obtained clues concerning the catalytic residues and mechanism of the fourth step of the FA elongation cycle by analyzing the mutants of Tsc13/TECR. In addition, we revealed the cooperative action of the enzymes catalyzing the fourth step (Tsc13) and the first step (Elo2 and Elo3) in the FA elongation cycle. Further studies are needed to elucidate the interactions and mutual regulation between Tsc13/TECR and other FA elongation cycle proteins, as well as the pathophysiological functions of TECR in various mammalian tissues.

## Experimental procedures

### Yeast strains and media

The *Saccharomyces cerevisiae* strains used in this study were all derived from BY4741 (*MAT****a*** *his3*Δ*1 leu2*Δ*0 met15*Δ*0 ura3*Δ*0*) (63). The *tsc13*Δ/*CERS5* cells ABY80 (BY4741, *tsc13*Δ*::LEU2*/pAB119 [*3×FLAG-CERS5*]) were as described previously (45). YTY8 (ABY80, *ELO2-6×HA::KanMX4*) and YTY9 (ABY80, *ELO3-6×HA::KanMX4*), in which *6×HA* tag was chromosomally fused with the 3′-termini of *ELO2* and *ELO3*, respectively, were generated *via* PCR-based gene modification as described elsewhere (64). Briefly, the DNA sequence containing the *6×HA* sequence and a *KanMX4* marker was amplified by PCR from the pYM14 plasmid (64) using the primer set containing the homologous region of *ELO2* or *ELO3* (ELO2-F/R or ELO3-F/R; Table 2), respectively. Each amplified fragment was used for homologous recombination of the 3′-terminal region of *ELO2* or *ELO3*.

**Table 2:** Primers used in this study

Primer	Nucleotide sequence
TSC13-F	5′-AGGATCCATGCCTATCACCATAAAAAGCCGCTC-3′ (*Bam*HI site underlined)
TSC13-R	5′-GACGAAGATGACGTTGTCAGCTCAAGC-3′
TSC13 G79A-F	5′-CATCAAAGATTTGGCTCCCCAAATTTCATGG-3′
TSC13 G79A-R	5′-CCATGAAATTTGGGGAGCCAAATCTTTGATG-3′
TSC13 E91A-F	5′-GTCTTCTTTTGTGCGTATTTGGGTCCAGTC-3′
TSC13 E91A-R	5′-GACTGGACCCAAATACGCACAAAAGAAGAC-3′
TSC13 Y92A-F	5′-GTCTTCTTTTGTGAGGCTTTGGGTCCAGTCTTG-3′
TSC13 Y92A-R	5′-CAAGACTGGACCCAAAGCCTCACAAAAGAAGAC-3′
TSC13 G94A-F	5′-CTTTTGTGAGTATTTGGCTCCAGTCTTGGTTCAC-3′
TSC13 G94A-R	5′-GTGAACCAAGACTGGAGCCAAATACTCACAAAAG-3′
TSC13 K140A-F	5′-GGACATTATGGAGCGAGATTATTTGAAACC-3′
TSC13 K140A-R	5′-GGTTTCAAATAATCTCGCTCCATAATGTCC-3′
TSC13 T145A-F	5′-GAGATTATTTGAAGCCTTATTTGTTCAC-3′
TSC13 T145A-R	5′-GTGAACAAATAAGGCTTCAAATAATCTC-3′
TSC13 T155A-F	5′-CAATTCTCTTTAGCTGCTATGCCAATTTTCAAC-3′
TSC13 T155A-R	5′-GTTGAAAATTGGCATAGCAGCTAAAGAGAATTG-3′
TSC13 N160A-F	5′-CTATGCCAATTTTCGCCCTGTTCAAAAATTG-3′
TSC13 N160A-R	5′-CAATTTTTGAACAGGGCGAAAATTGGCATAG-3′
TSC13 K163A-F	5′-CAATTTTCAACCTGTTCGCAAATTGTTTCCATTAC-3′
TSC13 K163A-R	5′-GTAATGGAAACAATTTGCGAACAGGTTGAAAATTG-3′
TSC13 N164A-F	5′-CAACCTGTTCAAAGCTTGTTTCCATTACTGG-3′
TSC13 N164A-R	5′-CCAGTAATGGAAACAAGCTTTGAACAGGTTG-3′
TSC13 C165A-F	5′-CCTGTTCAAAAATGCTTTCCATTACTGGG-3′
TSC13 C165A-R	5′-CCCAGTAATGGAAAGCATTTTTGAACAGG-3′
TSC13 Y168A-F	5′-CAAAAATTGTTTCCATGCCTGGGTTCTAAGCGG-3′
TSC13 Y168A-R	5′-CCGCTTAGAACCCAGGCATGGAAACAATTTTTG-3′
TSC13 Y178A-F	5′-CTCATTTCATTCGGTGCCTTTGGCTACGGCTTC-3′
TSC13 Y178A-R	5′-GAAGCCGTAGCCAAAGGCACCGAATGAAATGAG-3′
TSC13 Y221A-F	5′-GGAACTTTTATTGCGCCATTAAATTGCGCC-3′
TSC13 Y221A-R	5′-GGCGCAATTTAATGGCGCAATAAAAGTTCC-3′
TSC13 Y256A-F	5′-GTTGCTCCCAACGCTACTTTTGAAGTTTGG-3′
TSC13 Y256A-R	5′-CCAAACTTCAAAAGTAGCGTTGGGAGCAAC-3′
TECR E94A-F	5′-GACGGTCTTCCTAACAGCGTACGCGGGGCCCC-3′
TECR E94A-R	5′-GGGGCCCCGCGTACGCTGTTAGGAAGACCGTC-3′
TECR T155A-F	5′-CGCTTCTCCCATGGCGCGATGCCTTTGCGCAAC-3′
TECR T155A-R	5′-GTTGCGCAAAGGCATCGCGCCATGGGAGAAGCG-3′
TECR Y168A-F	5′-GAACTGCACCTACGCGTGGGGCTTCGCCGCGTG-3′
TECR Y168A-R	5′-CACGCGGCGAAGCCCCACGCGTAGGTGCAGTTC-3′
TECR Y177A-F	5′-CGCCGCGTGGATGGCCGCGTACATCAATCACCC-3′
TECR Y177A-R	5′-GGGTGATTGATGTACGCGGCCATCCACGCGGCG-3′
TECR Y248A-F	5′-GTGTCCTGCCCCAACGCGACCTACGAGGTGGGG-3′
TECR Y248A-R	5′-CCCCACCTCGTAGGTCGCGTTGGGGCAGGACAC-3′
ELO2-F1	5′-TTAATGAGTATGTTAACGTTGACTTGAAAAACGTTCCTACTCCATCTCCATCACCAAAACCTCAACACAGAAGAAAAAGGTCCGGTTCTGCTGCTAGATACCCATACG-3′
ELO2-F2	5′-TACTTATCACCATGGCGCTACTGCC-3′
ELO2-R1	5′-GATAATAGTAACAATAGATCCACTAAGAAAAAACGAATATACGAAAGTTGAATACTTAAATTCTTTCAAAGATTTTACACGAATCTTTTTATTGTCAGTACTGA-3′
ELO2-R2	5′-ATATTGCATTTGCTTCAAATTATGG-3′
ELO3-F1	5′-CTGAAGTTTCCGGCTCCGTTGCATCCGGTTCTTCTACTGGTGTCAAGACCTCTAACACCAAGGTCTCTTCCAGGAAAGCTTCCGGTTCTGCTGCTAGATACCCATACG-3′
ELO3-F2	5′-CCATCACGGTGCCACCGCTTTGTTGTG-3′
ELO3-R1	5′-CGTACATATTTAATATAAAGAAAATGTTAACATTTAATTTTTTTCTTTTTCATTCGCTGTCAAAAATTCTCGCTTCCTATGAATCTTTTTATTGTCAGTACTGA-3′
ELO3-R2	5′-CCAATACTTAGATAATGATGCTGGGCCG-3′

Yeast cells were grown in synthetic complete (SC) medium without histidine or uracil (SC –His –Ura) at 30 °C. The medium contained 0.67% yeast nitrogen base (Merck), 2% D-glucose, 1% Complete Supplement Mixture –HIS –LEU –TRP –URA (MP Biomedicals), 100 mg/L leucine, and 20 mg/L tryptophan. Cells grown overnight were diluted to *A*_600_ = 0.3 (∼0.4 × 10^7^ cells/ml), grown to *A*_600_ = ∼1.0, collected, and used for experiments described below.

### Plasmids

The yeast vector pAKNF426 (*URA3* marker, *2 μ* origin) is designed to express a 5′-terminally *3*×*FLAG*-tagged gene under the control of the *TDH3* (glyceraldehyde-3-phosphate dehydrogenase) promoter. The pHN62 plasmid encoding *3×FLAG-TSC13* was created as follows. A DNA fragment containing the coding sequence of *TSC13* + 340 bp 3′-untranslated region was amplified from yeast genomic DNA *via* PCR using primers TSC13-F and TSC13-R (Table 2) and was cloned into the TA cloning vector pGEM-T Easy (Promega), generating the pMY52 plasmid. The pHN62 was then constructed by transferring the *Bam*HI-*Not*I fragment of the pMY52 plasmid into the pAKNF426 vector.

Plasmids encoding the *3×FLAG-TSC13* mutants (pHN47 [*G79A*], pHN48 [*E91A*], pHN49 [*Y92A*], pHN50 [*G94A*], pHN51 [*K140A*], pHN52 [*T145A*], pHN53 [*T155A*], pHN54 [*N160A*], pHN55 [*K163A*], pHN56 [*N164A*], pHN57 [*C165A*], pHN58 [*Y168A*], pHN59 [*Y179A*], pHN60 [*H221A*], and pHN61 [*Y256A*]) were constructed by introducing each mutation into the pMY52 plasmid using appropriate primer sets (Table 2) and the QuikChange site-directed mutagenesis kit (Agilent Technologies) according to the manufacturer’s instruction, followed by transfer of the mutated *TSC13* into the pAKNF426 vector. Plasmids encoding the double mutants of *3×FLAG-TSC13* (pRK22 [*Y168A/Y179A*], pRK23 [*Y179A/Y256A*], and pRK24 [*Y168A/Y256A*]) were similarly produced, using the pMY52 derivative with the *Y168A* or *Y179A* mutation as a template. The pRK25 plasmid, which expresses *3*×*FLAG*-tagged human *TECR* in yeast, was created by transferring *TECR* from the pCE-puro 3×FLAG-TER plasmid (29) to the pAKNF426 vector. Plasmids encoding the *TECR* mutants (pRK26 [*E94A*], pRK27 [*T155A*], pRK28 [*Y168A*], pRK29 [*Y177A*], and pRK30 [*Y248A*]) were constructed as described above.

### Immunoblotting

Immunoblotting was performed as described previously (65). Rabbit anti-FLAG polyclonal antibody (1/2000 dilution) (66), mouse anti-FLAG mAb (M2; 1 μg/ml; Merck), rabbit anti-Pgk1 polyclonal antibody (1/500 dilution) (67), and anti-HA mAb (HA3F10, 1/1000 dilution; Merck) were used as primary antibodies. Anti-rabbit IgG, HRP-linked F(ab′)_2_ fragment (1:7500 dilution; Cytiva), anti-mouse IgG, HRP-linked F(ab′)_2_ fragment (1:7500 dilution; Cytiva), and anti-rat IgG, HRP-linked whole antibody (1:6000 dilution; Cytiva) were used as secondary antibodies. Chemiluminescence reactions were performed using chemiluminescence reagents (100 mM Tris-HCl [pH 8.5], 0.2 mM *p*-coumaric acid, 2.5 mM luminol, 0.02% hydrogen peroxide) or Western Lightning Plus-ECL (PerkinElmer Life Sciences), followed by detection using X-ray film (Kodak) or quantification using Amersham Imager 600 (Cytiva).

### Preparation of total cell lysates

Approximately 0.8 × 10^7^ yeast cells were collected by centrifugation, suspended in 1 ml solution containing 0.25 M NaOH and 1% 2-mercaptoethanol, and incubated for 15 min on ice. Cells were then treated with 70 μl 100% trichloroacetic acid (w/v) and incubated for 10 min on ice. After washing with 500 μl 1 M Tris, samples were suspended in 1 × SDS sample buffer (62.5 mM Tris-HCl [pH 6.8], 2% SDS, 10% glycerol), mixed vigorously for 5 min, heated to 37 °C for 5 min, and mixed vigorously for another 5 min. A portion of the samples was subjected to protein quantification using the BCA Protein assay kit (Thermo Fischer Scientific), according to the manufacturer’s instruction. The remaining samples were treated with bromophenol blue (final concentration, 0.01%) and 2-mercaptoethanol (final concentration, 5%), incubated at 37 °C for 5 min, separated *via* SDS-PAGE, and subjected to immunoblotting using rabbit anti-FLAG polyclonal antibody and rabbit anti-Pgk1 polyclonal antibody as primary antibodies.

### Preparation of membrane fractions

Approximately 1.3 × 10^8^ yeast cells were suspended in 1 ml buffer A (100 mM Tris-HCl [pH 9.4], 40 mM 2-mercaptoethanol) and incubated at room temperature for 10 min. After centrifugation (4 °C, 2000*g*, 3 min), cells were suspended in 2 ml buffer B (50 mM Tris-HCl [pH 7.5], 1.2 M sorbitol) and incubated with 0.1 mg zymolyase (Zymolyase 100T; Nacalai Tesque) at 30 °C for 30 min to disrupt the cell walls. To remove the zymolyase, the cell suspensions were loaded into 2 ml of 1.8 M sorbitol and centrifuged (4 °C, 2000*g*, 5 min). Cell pellets were then suspended in lysis buffer (50 mM Hepes/NaOH [pH 6.8], 150 mM NaCl, 10% glycerol, 1 mM DTT, 1 mM PMSF, 1 × protease inhibitor cocktail [Complete EDTA-free, Merck]), lysed by sonication, and centrifuged (4 °C, 2000*g*, 3 min). The resulting supernatants (total cell lysates) were subjected to ultracentrifugation (4 °C, 100,000*g*, 30 min), and the pellets (membrane fractions) were suspended in lysis buffer by sonication.

### *In vitro* FA elongation assays

The *in vitro* FA elongation assay using radiolabeled malonyl-CoA was performed essentially as described previously (20). Briefly, membrane fractions (10 μg) were incubated with 27 μM (75 nCi) [^14^C]malonyl-CoA (American Radiolabeled Chemicals), 20 μM C18:0-CoA (Merck), and 1 mM NADPH (Merck) at 37 °C for 30 min. After the reaction, samples were treated with alkaline to hydrolyze acyl-CoAs to FAs, neutralized, extracted, and dried. Lipids were separated *via* TLC (Silica gel 60 TLC plate, Merck) with hexane/diethyl ether/acetic acid = 30:70:1 (v/v) and detected using an imaging analyzer BAS-2500 (FUJIFILM Wako Pure Chemical Corporation).

The *in vitro* FA elongation assay using stable isotope-labeled malonyl-CoA was performed as follows. Membrane fractions (10 μg) were incubated with 100 μM [^13^C]malonyl-CoA (Merck), 10 μM C18:0-CoA, and 1 mM NADPH in 50 μl reaction solution (50 mM Hepes-NaOH [pH 6.8], 150 mM NaCl, 1 mM MgCl_2_, 0.5 mM CaCl_2_, 200 μg/ml cerulenin, 10% glycerol, 1 mM DTT, 1 mM PMSF, 1 × protease inhibitor cocktail) at 37 °C for 30 min. After the reaction, 1 pmol *d*_31_-palmitic acid (Cayman Chemical) was added to the samples as an internal standard. Samples were then incubated with 25 μl 75% KOH (w/v) and 50 μl ethanol at 70 °C for 1 h to hydrolyze acyl-CoAs, neutralized with 19.1 μl 26.3 M formic acid and 50 μl ethanol, and mixed vigorously with 700 μl hexane for phase separation. After centrifugation (20,400*g*, room temperature, 3 min), the upper layer (organic phase) was collected and dried. The FAs obtained were derivatized with *N*-(4-aminomethylphenyl) pyridinium (AMP) amide using the AMP^+^ Mass Spectrometry Kit (Cayman Chemical) according to the manufacturer’s manual and subjected to LC-MS/MS analyses as described below.

### Lipid extraction from yeast

Approximately 1.3 × 10^7^ yeast cells were suspended in 100 μl extraction solution (ethanol/water/diethyl ether/pyridine/28% ammonia = 15:15:5:1:0.018 [v/v]) containing 5 pmol of *N*-(2′-(*R*)-hydroxypalmitoyl(*d*_9_))-D-*ribo*-phytosphingosine (Avanti Polar Lipids) as an internal standard and incubated at 60 °C for 15 min. After centrifugation (2300*g*, room temperature, 2 min), the supernatant was collected. The pellet was subjected to the lipid extraction procedure again, and the supernatant was added to the previous one. Pooled lipid extracts were incubated with 700 μl of chloroform/methanol = 1:2 (v/v) and 37.5 μl of 3 M KOH in methanol at 37 °C for 30 min to hydrolyze glycerolipids, neutralized with 22.5 μl of 5 M formic acid, and mixed vigorously with 250 μl of chloroform and 250 μl of water for phase separation. After centrifugation (2600*g*, room temperature, 3 min), the lower layer (organic phase) was collected and dried. The lipids obtained were dissolved in 1 ml of chloroform/methanol = 1:2 (v/v) and subjected to ceramide measurement *via* LC-MS/MS as described below.

### *d*_7_-Sphingosine labeling assay

Cells were labeled with 1 μM *d*_7_-sphingosine (Avanti Polar Lipids) at 30 °C for 30 min. Lipids were extracted from yeast, treated with alkali, neutralized, and subjected to phase separation as described above, except that 50 pmol *d*_31_-palmitic acid (Avanti Polar Lipids) was used as an internal standard. The organic phase was then collected and dried. The FAs were derivatized with AMP amide using the AMP^+^ Mass Spectrometry Kit (Cayman Chemical) and subjected to LC-MS/MS analyses as described below.

### LC-MS/MS analyses

The LC-MS/MS analyses were conducted using an LC-coupled tandem quadrupole mass spectrometer (Xeno TQ-S; Waters). The LC separation was performed using a reversed-phase column (ACQUITY UPLC CSH C18 column; particle size 1.7 μm; column diameter 2.1 mm, column length 100 mm; Waters) at 55 °C at a flow rate of 0.3 ml/min. The AMP amide-derivatized FAs were separated in a binary gradient system with mobile phase A (acetonitrile/water containing 5 mM ammonium formate = 3:2 [v/v]) and mobile phase B (isopropanol/acetonitrile containing 5 mM ammonium formate = 9:1 [v/v]) as follows: 0 min, 10% B; 0 to 6 min, gradient to 40% B; 6 to 15 min, gradient to 70% B; 15 to 18 min, gradient to 100% B; 18 to 23 min, 100% B; 23 to 23.1 min, gradient to 10% B; 23.1 to 25 min, 10% B. Separation of ceramides *via* LC was performed as described previously (68). Ionization of lipids was performed *via* electrospray ionization under the conditions described previously (69). Lipids were separated and detected *via* MS/MS in multiple reaction monitoring mode. The *m/z* values for the precursor ions (Q1), product ions (Q3), and collision energies are listed in Table 3 (for AMP amide-derivatized FAs) or were as described previously (for ceramides) (69). Data were analyzed using MassLynx software (Waters). The quantification of each lipid was calculated from the peak area relative to that of the corresponding internal standard.

**Table 3:** MS/MS settings for AMP amide-derivatized FAs

FA	Precursor ion (Q1)	Product ion (Q3)	Collision energy (eV)
	[M+H]^+^		
[^13^C]C20:0 FA	481.4	183.0	50
[^13^C]C22:0 FA	511.4	183.0	50
[^13^C]C24:0 FA	541.4	183.0	50
[^13^C]C26:0 FA	571.4	183.0	50
*Trans*-2-[^13^C]C20:1 FA	479.4	183.0	50
*Trans*-2-[^13^C]C22:1 FA	509.4	183.0	50
*Trans*-2-[^13^C]C24:1 FA	539.4	183.0	50
*Trans*-2-[^13^C]C26:1 FA	569.4	183.0	50
*d*_7_-C16:0 FA	430.4	183.0	50
*d*_7_-*Trans*-2-C16:1 FA	428.4	183.0	50
*d*_7_-*Cis*-9-C16:1 FA	428.4	183.0	50
*d*_31_-C16:0 FA^a^	454.4	183.0	50

a Internal standard.

### Indirect immunofluorescence microscopy

Indirect immunofluorescence microscopy was performed as described previously (70), using rabbit anti-FLAG polyclonal antibody (1/1000 dilution) (66) and mouse anti-HA mAb (HA-7; 1/400 dilution; Merck) as primary antibodies and Alexa Fluor 488-conjugated anti-rabbit IgG antibody and Alexa Fluor 594-conjugated anti-mouse IgG antibody (1/200 dilution each; Thermo Fisher Scientific) as secondary antibodies. Cells were mounted on glass microscope slides with ProLong Gold Antifade reagent (Thermo Fisher Scientific) and observed under a Leica DM5000B microscope (Leica Microsystems).

### Co-immunoprecipitation

Approximately 2.5 × 10^8^ yeast cells were suspended in 500 μl of immunoprecipitation buffer (50 mM Hepes/NaOH [pH 7.5], 0.2 M sorbitol, 150 mM NaCl, 1 mM DTT, 1 mM PMSF, 1 × protease inhibitor cocktail) and transferred to plastic tubes containing glass beads (acid-washed, 425–600 μm; Merck). Cells were disrupted by vigorous mixing (4000 rpm, 4 °C, 1 min) using a Micro Smash MS-100 (TOMY Seiko). After centrifugation (2300*g*, 4 °C, 3 min), the supernatant was collected. The pellet was suspended in 500 μl of immunoprecipitation buffer and subjected to cell disruption again. The supernatant was collected and mixed with the previous one, and the pooled supernatant was ultracentrifuged (100,000*g*, 4 °C, 30 min). The pellet obtained (membrane fraction) was suspended in 150 μl of immunoprecipitation buffer by sonication, of which a 120 μl aliquot was diluted with 528 μl immunoprecipitation buffer and treated with 72 μl 10% Triton X-100 for solubilization. After rotation at 4 °C for 1 h, samples were subjected to ultracentrifugation (100,000*g*, 4 °C, 30 min). The supernatant (solubilized membrane fraction) was collected and incubated with an anti-FLAG M2 affinity gel (bed volume 15 μl; Merck) at 4 °C overnight with rotation. The gels were washed twice with 0.5 ml immunoprecipitation buffer containing 0.1% Triton X-100, and the bound proteins were eluted from the gels by suspending them in 32.5 μl 2 × SDS sample buffer and incubating them at 37 °C for 5 min. After centrifugation, the supernatant (immunoprecipitation fraction) was collected, separated by SDS-PAGE, and detected *via* immunoblotting using mouse anti-FLAG M2 mAb and rat anti-HA HA3F10 mAb as primary antibodies.

### Amino acid mapping

The structural model of Tsc13 was obtained from the AlphaFold Protein Structure Database (https://alphafold.ebi.ac.uk; UniProt ID Q99190), and amino acid residues whose activity is reduced by Ala-substitution were mapped using PyMOL software (version 2.5.2; Schrödinger).

## Data availability

All data generated or analyzed during this study are contained within the article.

## Supporting information

This article contains supporting information.

## Conflict of interest

The authors declare that they have no conflicts of interest with the contents of this article.
