# Supplementary material for: Catalytic mechanism of trans-2-enoyl-CoA reductases in the fatty acid elongation cycle and its cooperative action with fatty acid elongases
Source: J Biol Chem. 2024 Jan 13;300(2):105656. doi: 10.1016/j.jbc.2024.105656 (PMC10864336; doi:10.1016/j.jbc.2024.105656)
Supplement: Supporting Figure S1 [file mmc1.pdf]

```
H. sapiens        1 MKHYEVEILDAKTR-EKLCFLDKVEPHATIAEIKNLFTK-THPQWYPARQ--SLRLDPKGKS----LKDE  62
M. musculus       1 MKHYEVEIRDAKTR-EKLCFLDKVEPQATISEIKTLFTK-THPQWYPARQ--SLRLDPKGKS----LKDE  62
R. norvegicus     1 MKHYEVEIRDAKTR-EKLCFLDKVEPQATISEIKTLFTK-THPQWYPARQ--SLRLDPKGKS----LKDE  62
B. taurus         1 MKHYEVEILDAKTR-EKLCFLDKVEPQATIAEIKNLFTK-THPQWYPARQ--SLRLDPKGKS----LKDE  62
G. gallus         1 MKHYEVEILDAKTR-EKLCFLDKVEPQATIAEIKNLFTK-THPQWYPARQ--SLRLEPKGRS----LKDE  62
X. laevis         1 MKHHEVEILDAKTR-EKLCFLDKVEPHATIADIKNMFTK-SHPQWYPARQ--SLRLDPKGRS----LKDE  62
D. rerio          1 MKHYEVEILDAKTK-DKLCFLDKVEPNATIGEIKSMFHK-SHPQWYPARQ--SIRLDPKGKS----LKDE  62
D. melanogaster   1    MELEIVNAKNS-KPYGKVKVPSAATPISDLRALIHKSLKQTPNANRQ--SLRLELKGKS----LKDT  60
A. aegypti        1    MEVEILDAKNS-KPIGKCRVQG-DTSIKALKNEVQN-MKKSLTVHRQ--ALRLEPRGKI----LKDS  58
B. malayi         1 MTSIAVEVFDAKNINKSIAYLENLS-------------------LPINQI--ALRLDAKGKN----LKDD  45
C. elegans        1  MSGILEVYDAKRTDNLIITLEGISGSETIKAIKKRIAQ-KKLKLTEERQ--ALRVEPKGKP----LADD  62
A. thaliana       1    MKVTVVSRSGR-EVLKAPLDLPDSATVADLQEAFHK-RAKKFYPSRQRLTLPVTPGSKDKPVVLNSK  65
S. cerevisiae     1  MPITIKSRSKGLRDTEIDLSKKPTLDDVLKKISANNHN-----ISKYRIRLTYKKESKQVP----VISE  60
                         .                                                            .

                                                        TH1
H. sapiens       63 DVLQKLPVGT--TATLYFRDLGAQISWVTVFLTEYAGPLFIYLLFYFR---VPFIYGHKYDFTSSRHTVV 127
M. musculus      63 DVLQKLPVGT--TATLYFRDLGAQISWVTVFLTEYAGPLFIYLLFYFR---VPFIYGRKYDFTSSRHTVV 127
R. norvegicus    63 DVLQKLPVGT--TATLYFRDLGAQISWVTVFLTEYAGPLFIYLLFYFR---VPFIYGRKYDFTSSRHTVV 127
B. taurus        63 DVLQKLPVGT--TATLYFRDLGAQISWVTVFLTEYAGPLFIYLLFYFR---VPFIYGRKYDFTSSRHTVV 127
G. gallus        63 DVLQSLPVGT--TATFYFRDLGAQISWVTVFLTEYAGPLLIYLLFYFR---VPFLYGPRYDFTASRHAVV 127
X. laevis        63 DILQNLPVGT--TATLYFRDLGAQISWVTVFLTEYAGPLVIYLLFYFR---VPFIYGPKYDFITSRHSVV 127
D. rerio         63 DVLQHLPVGT--TATFYFRDLGAQISWVTVFLTEYAGPLLIYLMFYFR---VPFIYAPKYDFTTSKHWVV 127
D. melanogaster  61 DTLETLSLRS--GDKVYVKDLGPQIGWKTVFLAEYAGPLIVYLIFYFR---PELIYG-KSAT-LPISLTT 123
A. aegypti       59 DTLQSLNFTS--GGKLYVKDLGPQISWKGVFLAEYAGPIFVYMIFYQR---PSLIYG-SAAA-NPVSLTA 121
B. malayi        46 LVVLDLNLPSK-GAHLYIRVLGPQIGWKTVFLLEYIGPLVIYPIFYLR---PTEIYG-PDASRYPMSYGV 110
C. elegans       63 QKLSDLGLSSQ-KAVLYVRDLGPQIAWKTVFMAEYAGPLFVYPLFYLR---PTFIYG-QAAVNATMHPAV 127
A. thaliana      66 KSLKEYCDGNNNSLTVVFKDLGAQVSYRTLFFFEYLGPLLIYPVFYYF---PVYKFL-GYGEDCVIHPVQ 131
S. cerevisiae    61 SFFQEEADDS---MEFFIKDLGPQISWRLVFFCEYLGPVLVHSLFYYLSTIPTVVDRWHSASSDYNPFLN 127
                                      . ** *.    .*  ** **. .   **

                             TH2                            TH3
H. sapiens      128 HLACICHSFHYIKRLLETLFVHRFSHGTMPLRNIFKNCTYYWGFAAWM--AYYIN-------HPLYTPPT 188
M. musculus     128 HLACMCHSFHYIKRLLETLFVHRFSHGTMPLRNIFKNCTYYWGFAAWM--AYYIN-------HPLYTPPT 188
R. norvegicus   128 HLACMCHSFHYIKRLLETLFVHRFSHGTMPLRNIFKNCTYYWGFAAWM--AYYIN-------HPLYTPPT 188
B. taurus       128 HLACICHSFHYIKRLLETLFVHRFSHGTMPLRNIFKNCTYYWGFAAWM--AYYIN-------HPLYTPPT 188
G. gallus       128 HLACACHSFHYIKRLLETLFVHRFSHGTMPLRNIFKNCTYYWGFAAWM--AYYIN-------HPLYTPPA 188
X. laevis       128 HLACICHSFHYIKRLLETLFVHRFSHGTMPFRNIFKNCMYYWGFAAWM--AYYIN-------HPLYTPPT 188
D. rerio        128 HLACMCHSFHYVKRLLETLFVHRFSHGTMPLRNIFKNCTYYWGFAAWM--AYYIN-------HPLYTPPI 188
D. melanogaster 124 HIAAGCYTVHYVKRLLETIFVHRFSHATMPLRNLFKNCTYYWGFTAYV--SYHVN-------HPQFTSPC 184
A. aegypti      122 NVAALCWIAHYAKRLLETLFVHRFSHATMPLRNLFKNCTYYWAFAGYV--AYHVN-------HPLFTEPS 182
B. malayi       111 KLALVCWSFHYAKRLLETLFVHRFSNATMPLRNIFKNCGYYWAFTAFV--SYFIN-------HPLYTLPY 171
C. elegans      128 QIAFFAWSFHYAKRLFETQFIHRFGNSTMPQFNLVKNCSYYWGFAAFV--AYFVN-------HPLFTPPA 188
A. thaliana     132 TYAMYYWCFHYFKRILETFFVHRFSHATSPIGNVFRNCAYYWSFGAYI--AYYVN-------HPLYTPVS 192
S. cerevisiae   128 RVAYFLILGHYGKRLFETLFVHQFSLATMPIFNLFKNCFHYWVLSGLISFGYFGYGFPFGNAKLFKYYSY 197
                      *      *. **  ** *.*.*   * *  *. .**  **         *

                                        TH4                                    TH5
H. sapiens      189 YGAQQVKLALAIFVICQLGNFSIHMALRDLR-PAGSKTRKIPYPTKNPFTWLFLLVSCPNYTYEVGSWIG 257
M. musculus     189 YGVQQVKLALAVFVICQLGNFSIHMALRDLR-PAGSKTRKIPYPTKNPFTWLFLLVSCPNYTYEVGSWIG 257
R. norvegicus   189 YGVQQVKLALAIFVICQLGNFSIHMALRDLR-PAGSKTRKIPYPTKNPFTWLFLLVSCPNYTYEVGSWIG 257
B. taurus       189 YGAQQVKLALAIFVICQLGNFSIHMALRDLR-PAGSKTRKIPYPTRNPFTWLFLLVSCPNYTYEVGSWIG 257
G. gallus       189 YGDEQVKLALAVFLFCQLGNFSIHVALRNLR-PAGSKTRKIPYPTRNPFTWLFLLVSCPNYTYEVGSWIG 257
X. laevis       189 YGENQVKLAVIIFLFCQLGNFSIHIALRNLR-PAGSKTRKIPFPTRNPFTWLFLLVSCPNYTYEVGSWIG 257
D. rerio        189 YGEQQIRLALTVFLFCQIGNFSIHIALRNLR-PPGSKTRKIPYPTKNPFTWIFLLVSCPNYTYELGSWLG 257
D. melanogaster 185 M--CTVWAALGAFALCELGNFSVHIALRNLR-PPGTKVRKIPVADANPLTKLFNLVSCPNYTYEIGAWVS 251
A. aegypti      183 T--AVMYAGLAGFIVSELGNFSIHMLLRNLR-PAGSTVRKIPKPDGNPLTQLFNFVSCPNYTYEFLSWLS 249
B. malayi       172 FGFVQVATGLIGFVICEFGNLSVHLLLRNLR-PSGTKVRKIPIPDINPMTLMFHFVSCPNYTYEVGSWLW 240
C. elegans      189 FGDLQVYFGLAGFVISEFGNLSIHILLRNLR-PAGTRERRIPKPDGNPLSLLFNYVSCPNYTYEVASWIF 257
A. thaliana     193 D--LQMKIGFGFGLVCQVANFYCHILLKNLRDPSGAGGYQIPRG------FLFNIVTCANYTTEIYQWLG 254
S. cerevisiae   198 LKLDDLSTLIGLFVLSELWNFYCHIKLRLWG--DYQKKHGNAKIRVPLNQGIFNLFVAPNYTFEVWSWIW 265
                         .         .   *   *. *.                        *      *** *   *.

                                              TH6
H. sapiens      258 FAIMTQC-LPVALFSLVGFTQMTIWAKGKHRSYLKEFR------DYPPLRMPIIPFLL 308
M. musculus     258 FAILTQC-VPVALFSLVGFTQMTIWAKGKHRSYLKEFR------DYPPLRMPIIPFLL 308
R. norvegicus   258 FAIMTQC-VPVALFSLVGFTQMTIWAKGKHRSYLKEFR------DYPPLRMPIIPFLL 308
B. taurus       258 FAIMTQC-LPVALFSLVGFTQMTIWAKGKHRSYLKEFR------DYPPLRMPIIPFLL 308
G. gallus       258 FTIMTQC-LPVALFSLVGFVQMAIWAQGKHRSYLREFR------DYPPLRSPIVPFLL 308
X. laevis       258 FAIMTQC-FPVALFSLVGFIQMTIWAKGKHRSYLKEFR------DYPPLRSPIVPFFL 308
D. rerio        258 FTLMTQC-LPVAFFTLVGFIQMTVWAKGKHRSYLKEFR------DYPTLRSPILPFIL 308
D. melanogaster 252 FSVLTSC-LAAYLFAFAGAFQMTVWALAKHRNYRKEFK------DYPRQRRSIFPFVL 302
A. aegypti      250 FSLMTTC-VPALLFTAAGMYQMTVWAIGKHKNYKKDFK------DYPKGRKAILPFVI 300
B. malayi       241 FSYMTQS-LPALIFTFAGFLQMAIWAKDKHRNYRREFP------NYPKHRRAMIPFVF 291
C. elegans      258 FSIMVQS-LPAIIFTTAGFAQMAIWAQGKHRNYLKEFP------DYPKNRKAIVPFVL 308
A. thaliana     255 FNIATQT-IAGYVFLAVAALIMTNWALGKHSRLRKIFDGKDGKPKYPRRWVILPPFL  310
S. cerevisiae   266 FTFVFKFNLFAVLFLTVSTAQMYAWAQKKNK-------------KYHTRRAFLIPFVF 310
                    *            *       *  **  *.               *      . **
```

Figure S1. Sequence alignment of Tsc13/TECR orthologs. The amino acid sequence alignment of Tsc13/TECR orthologs from 13 species are shown. Amino acid residues mutated in this study are indicated in yellow. The predicted THs in the structural model of Tsc13 obtained from the AlphaFold Protein Structure Database is shown in gray. The species and GenBank accession numbers for each amino acid sequence are as follows: *Homo sapiens*, NP_612510.1; *Mus musculus*, NP_598879.1; *Rattus norvegicus*, NP_612558.1; *Bos taurus*, NP_001029920.1; *Gallus gallus*, XP_046760717.1; *Xenopus laevis*, NP_001085733.1; *Danio rerio*, NP_958456.1; *Drosophila melanogaster*, NP_647836.2; *Aedes aegypti*, XP_001653426.2; *Brugia malayi*, XP_001899974.2; *Caenorhabditis elegans*, NP_495430.1; *Arabidopsis thaliana*, NP_191096.1; *Saccharomyces cerevisiae*, NP_647836.2.
